# Escape of TLR5 Recognition by *Leptospira* spp.: A Rationale for Atypical Endoflagella

**DOI:** 10.3389/fimmu.2020.02007

**Published:** 2020-08-11

**Authors:** Marion Holzapfel, Delphine Bonhomme, Julie Cagliero, Frédérique Vernel-Pauillac, Martine Fanton d’Andon, Sophia Bortolussi, Laurence Fiette, Cyrille Goarant, Elsio A. Wunder, Mathieu Picardeau, Albert I. Ko, Dirk Werling, Mariko Matsui, Ivo G. Boneca, Catherine Werts

**Affiliations:** ^1^Institut Pasteur, Unité Biologie et Génétique de la Paroi Bactérienne, Paris, France; ^2^CNRS, UMR 2001 Microbiologie Intégrative et Moléculaire, Paris, France; ^3^Institut National de la Santé et de la Recherche Médicale, Equipe Avenir, Paris, France; ^4^Sorbonne Paris Cité, Université de Paris, Paris, France; ^5^Institut Pasteur de Nouvelle Calédonie, Immunity and Inflammation Group, Institut Pasteur International Network, Noumea, France; ^6^Unité Histopathologie Humaine et Modèles Animaux, Institut Pasteur, Paris, France; ^7^Leptospirosis Research and Expertise Unit, Institut Pasteur International Network, Institut Pasteur de Nouvelle Calédonie, Noumea, France; ^8^Gonçalo Moniz Institute, Oswaldo Cruz Foundation, Brazilian Ministry of Health, Salvador, Brazil; ^9^Department of Epidemiology of Microbial Diseases, Yale School of Public Health, New Haven, CT, United States; ^10^Unité Biologie des Spirochètes, Institut Pasteur, Paris, France; ^11^Department of Pathobiology and Population Sciences, Royal Veterinary College, Hatfield, United Kingdom

**Keywords:** Leptospira, toll-like receptor, innate immunity, Flagelin genes, TLR5, mouse model

## Abstract

*Leptospira (L.) interrogans* are invasive bacteria responsible for leptospirosis, a worldwide zoonosis. They possess two periplasmic endoflagellae that allow their motility. *L. interrogans* are stealth pathogens that escape the innate immune recognition of the NOD-like receptors NOD1/2, and the human Toll-like receptor (TLR)4, which senses peptidoglycan and lipopolysaccharide (LPS), respectively. TLR5 is another receptor of bacterial cell wall components, recognizing flagellin subunits. To study the contribution of TLR5 in the host defense against leptospires, we infected WT and TLR5 deficient mice with pathogenic *L. interrogans* and tracked the infection by *in vivo* live imaging of bioluminescent bacteria or by qPCR. We did not identify any protective or inflammatory role of murine TLR5 for controlling pathogenic *Leptospira*. Likewise, subsequent *in vitro* experiments showed that infections with different live strains of *L. interrogans* and *L. biflexa* did not trigger TLR5 signaling. However, unexpectedly, heat-killed bacteria stimulated human and bovine TLR5, but did not, or barely induced stimulation via murine TLR5. Abolition of TLR5 recognition required extensive boiling time of the bacteria or proteinase K treatment, showing an unusual high stability of the leptospiral flagellins. Interestingly, after using antimicrobial peptides to destabilize live leptospires, we detected TLR5 activity, suggesting that TLR5 could participate in the fight against leptospires in humans or cattle. Using different *Leptospira* strains with mutations in the flagellin proteins, we further showed that neither FlaA nor Fcp participated in the recognition by TLR5, suggesting a role for the FlaB. FlaB have structural homology to *Salmonella* FliC, and possess conserved residues important for TLR5 activation, as shown by *in silico* analyses. Accordingly, we found that leptospires regulate the expression of FlaB mRNA according to the growth phase *in vitro*, and that infection with *L. interrogans* in hamsters and in mice downregulated the expression of the FlaB, but not the FlaA subunits. Altogether, in contrast to different bacteria that modify their flagellin sequences to escape TLR5 recognition, our study suggests that the peculiar central localization and stability of the FlaB monomers in the periplasmic endoflagellae, associated with the downregulation of FlaB subunits in hosts, constitute an efficient strategy of leptospires to escape the TLR5 recognition and the induced immune response.

## Introduction

Leptospires are spirochetal bacteria responsible for leptospirosis, a neglected reemerging zoonosis (1). Among the *Leptospira* genus, which includes more than 60 species and 300 different serovars, *Leptospira (L.) interrogans* gathers the most pathogenic strains (2). Rodents and other animals can carry leptospires asymptomatically in the lumen of proximal renal tubules, excrete the bacteria in their urine and contaminate the environment. Vertebrates get infected through skin or mucosa. In most cases, humans show either no symptoms or suffer from a flu-like mild disease, but may also show acute severe, potentially fatal, leptospirosis. Antibiotic treatments are efficient only if administered at the onset of symptoms. The high number of leptospiral serovars and strains complicates the diagnosis and impairs vaccine strategies.

*Leptospira* are motile bacteria able to swim very fast in viscous environments. They possess two endoflagella, one inserted at each pole of the bacteria, which do not protrude outside of the bacteria but are localized and rotate within the periplasmic space. As seen in other spirochetes, the leptospiral genomes exhibit an atypically high number of structural flagellar genes, including four FlaB subunits with homology to FliC, the unique flagellin monomer forming the filament of *Salmonella* spp. The structure of the leptospiral filament and the roles of the different flagellar proteins and additional specific components of leptospires, such as the Fcp proteins (3–7), have been recently elucidated by high-resolution cryo-electron microscopy coupled to model building and crystallography analyses (8). These studies revealed that the leptospiral filament has an atypical flattened helical shape, and that the four FlaB subunits constitute the core of the flagellum, surrounded by two FlaA and two Fcp subunits forming a sheath (8).

The innate defense of the host relies, amongst other mechanisms, on activation of the complement system and on the recognition of microbe-associated molecular patterns (MAMPs) by immune receptors, such as the pattern recognition receptors (PRRs) families of Toll-like Receptor (TLR) and NOD-like receptor (NLR). After ligand recognition, TLRs and NLRs induce immune inflammatory responses that trigger cellular recruitment, ultimately leading to the destruction of microbes by phagocytes (9).

FliC, the prototypical bacterial flagellin, forms a hairpin-like structure with 4 connected domains designated D0, D1, D2, and D3, with both C and N termini associated in the D0 domain (10). The D2 and D3 domains are highly variable and support the antigenic diversity. FliC is recognized by different PRRs. TLR5 is expressed at the surface of cells and recognizes monomers of flagellin through the D1 domain, whereas in the cytosol FliC is recognized through the D0 domain by the NAIP inflammasome, which associates with the IPAF/NLRC4, a NOD-like receptor (11, 12). TLR5 is an essential innate immune receptor expressed in the kidney and, along with TLR4, important to control *Enterobacteria* (13). Moreover it is one of the very few TLRs able to recognize a protein agonist, conferring potent adjuvant properties, and helping adaptive immune responses (14).

We previously showed that *Leptospira* infection triggers the NLRP3 inflammasome, using the ASC adaptor. The results using ASCko mice reproduced the results obtained with the NLRP3ko mice and suggest that the contribution of other inflammasome receptors, such as the NAIP/NLRC4 would be minimal (15). We also showed that *L. interrogans* escapes recognition by human TLR4 (16) as well as murine and human NOD1 and NOD2 (17). In this work, we investigated whether leptospiral flagellins are either recognized by or also escape recognition by TLR5. Our results suggest that live pathogenic leptospires largely escape recognition by human and murine TLR5, although their FlaB subunits are able to signal through human TLR5. This suggests that the periplasmic localization of the flagella and the concealing of FlaB in the core of the filament contribute to avoiding the TLR5 recognition pathway.

## Materials and Methods

### Leptospiral Strains and Culture Conditions

Pathogenic *L. interrogans* serovar Icterohaemorrhagiae strain Verdun, *L. interrogans* serovar Copenhageni strain Fiocruz L1-130, *L. interrogans* serovar Manilae strain L495, and the saprophytic *L. biflexa* serovar Patoc strain Patoc I have been provided by the collection of the National Reference Center for Leptospirosis of the Institut Pasteur in Paris. The L495 derivative bioluminescent strain MFLum1 (18), the clinical isolate Fiocruz LV2756 and its non-mobile *fcpA* mutant (5), the *L. interrogans* Manilae *flaA2* mutant, as well as the *flaB4* mutant of *L. biflexa* Patoc have all been previously described (3, 19). The *L. biflexa fcpA* and *L. interrogans* Manilae *flaB1* mutants have been generated for this study by random mutagenesis (20).

Bacteria were grown in Ellinghausen-McCullough-Johnson-Harris (EMJH) medium (Bio-Rad) at 30°C without agitation and weekly passaged, counted using a Petroff-Hauser chamber and seeded at 5 × 10^6^ bacteria per mL (bact/mL). Bacteria in mid-log exponential phase (around 10^8^ bact/mL), and bacteria in stationary phase (around 1 to 5 × 10^9^ bact/mL) were harvested from 3–6-day old cultures and 10–14-day old cultures, respectively. Unless otherwise specified, experiments were performed with 1-week old cultures. The *L. biflexa* Patoc Patoc I strain was passaged twice a week by a 1/250 dilution and seeded at around 5 × 10^6^ bact/mL. For experiments conducted in New Caledonia, virulent *L. interrogans* serovar Icterohaemorrhagiae strain Verdun was cultured in EMJH medium at 30°C under aerobic conditions as previously described (21). For *in vitro flaB* gene expression assays, cultures of each *Leptospira* strain were seeded in triplicate at 5 × 10^6^ (Day 0). On Day 3 (exponential growth phase) and Day 14 (stationary growth phase), 5 × 10^8^ bacteria from each culture were harvested and centrifuged at 3,250 × *g* for 25 min, EMJH was discarded and bacteria were resuspended in 500 μL of RNAlater Buffer (Qiagen) for RNA stabilization, kept at room temperature for 2 h before conservation at −20°C until RNA extraction.

### *In vivo* Infection Experiments Using Leptospires

Male and female C57BL/6J mice (7- to 10-week old) were used in this study and were obtained from Janvier Labs (Le Genest, France). TLR5 deficient mice (TLR5KO) in a C57BL/6J background were bred at the Institut Pasteur Paris animal facility and were previously described (18). Outbred OF1 mice (*Mus musculus*) and golden Syrian hamsters (*Mesocricetus auratus*), initially obtained from Charles River Laboratories, were bred in the animal facility in Institut Pasteur in New Caledonia.

Infections of C57BL/6J mice with *L. interrogans* strains were conducted as described (22). Just before infection, bacteria were centrifuged at room temperature for 25 min at 3,250 × *g*, resuspended in endotoxin-free PBS. Infection in mice were done via the intraperitoneal route (IP) with sublethal doses (10^7^ bacteria in 200 μL of PBS) of pathogenic *L. interrogans*. Live imaging (IVIS) using the bioluminescent MFLum1 derivative of Manilae L595 has been described earlier (23) and recently reviewed (24). Animals were bled at the facial vein sinus (around 50–100 μl of blood, recovered in tubes coated with 20 μl of EDTA 100 mM). A drop of urine was retrieved upon first handling of mice. Animals were killed by cervical dislocation and organs frozen in liquid nitrogen before storage at −80°C or fixed in formaldehyde for histopathology.

Virulence of *L. interrogans* Icterohaemorrhagiae strain Verdun was maintained by cyclic passages in golden Syrian hamsters after intraperitoneal (IP) injection of the LD_100_ at 2 × 10^8^ leptospires before re-isolation from blood by cardiac puncture at 4.5 days post-infection, after euthanasia with CO_2_.

For *in vivo* study of *flaA* and *flaB* gene expression, 6–8-week old healthy animals (*n* ≥ 5 individuals per condition) were infected and experiments were carried out as previously described (21). Briefly, OF1 mice and hamsters were IP injected with 2 × 10^8^ virulent *L. interrogans* Icterohaemorrhagiae strain Verdun in 500–800 μL of EMJH medium, as recently reviewed (25). After euthanasia with CO_2_, whole blood was rapidly collected by cardiac puncture at 24 h p.i. and conserved in PAXgene blood RNA tubes (PreAnalytiX, Qiagen) for 2 h at room temperature to allow stabilization of total RNA before storage at −20°C until RNA extraction.

### Ethics Statement

Animal manipulations were conducted according to the guidelines of Animal Care following the EU Directive 2010/63 EU. All protocols were reviewed and approved (#2013-0034, and #HA-0036) by the Institut Pasteur ethic committee (CETEA #89) (Paris, France), the competent authority, for compliance with the French and European regulations on Animal Welfare and with Public Health Service recommendations.

### Histology and Immunohistochemistry

Transversal sections of kidneys were collected and fixed in formaldehyde 4% for at least 48 h at room temperature, embedded in paraffin, and 5 μm thick sections were stained with Hematoxylin-Eosin. Immunohistochemistry was performed on dewaxed sections as described (18). A rabbit polyclonal serum against the LipL21 (kindly provided by David Haake) was used (1/1000^e^). A Periodic Acid-Schiff (PAS) staining was also associated to the Lip21 immunolabeling to visualize the membranes and brush borders typical of proximal tubules.

### qPCR Quantification of Leptospiral DNA in Blood, Urine and Organs

The leptospiral load in blood, urine and organs was determined by quantitative real-time PCR (qPCR), as described (22). Total DNA from blood and urine (around 50 μL) was extracted using a Maxwell 16 automat with the Maxwell blood DNA and cell LEV DNA purification kits (Promega), respectively. DNA was extracted with the QIAamp DNA kit (Qiagen) from organs mechanically disrupted for 3 min at 4°C with metal beads using an automat (Labomodern). Primers and probe designed in the *lpxA* gene of *L. interrogans* strain Fiocruz L1-130 (4) were used to specifically detect pathogenic *Leptospira sp.* (22), using the *nidogen* gene for normalization in kidneys. qPCR reactions were run on a Step one Plus real-time PCR apparatus using the absolute quantification program (Applied Biosystems), with the following conditions for FAM-TAMRA probes: 50°C for 2 min, 95°C for 10 min, followed by 40 cycles with denaturation at 95°C for 15 s and annealing temperature 60°C for 1 min.

### Reverse and Real-Time Transcription PCR for Cytokine Gene Expression in Kidney

Total RNA was extracted from kidneys using the RNeasy mini kit (Qiagen) and RT-qPCR were performed as described (18). The sequences of primers and probes for IL10, RANTES, and IFNγ have already been described (10, 15). Data were analyzed according to the method of relative gene expression using the comparative cycle threshold (*Ct*) method also referred to as the 2^(–ΔΔCt)^ method. PCR data were reported as the relative increase in mRNA transcripts versus that found in kidneys from naive WT mice, corrected by the respective levels of Hypoxanthine phosphoribosyltransferase (HPRT) mRNA used as an internal standard.

### Total RNA Extraction and cDNA Synthesis for Leptospiral *fla* Genes

Total RNA from blood was extracted using a PAXgene blood RNA system from PreAnalytiX (Qiagen). Total RNA from virulent *Leptospira* (4 × 10^8^ bacteria) cultured *in vitro* at 30 and at 37°C in EMJH medium was also extracted using a High Pure RNA Isolation kit (Roche Applied Science) following the manufacturer’s recommendations. Total RNA samples were treated with DNase (Turbo DNA-Free kit; Ambion, Applied Biosystems) for elimination of residual genomic DNA. Before storage at −80°C, purified RNA was quantified by measurement of the optical density at 260 nm (OD_260_) using a NanoDrop 2000 spectrophotometer (Thermo Fisher Scientific), and the quality of nucleic acids was verified by measurement of the OD_260_/OD_280_ ratio. Then, 1 μg of total RNA was reverse transcribed using a Transcriptor First Strand cDNA synthesis kit (Roche Applied Science) and the provided random hexamer primers for the mix preparation, on a GeneAmp PCR system 9700 instrument (Applied Biosystems) with the following program: 10 min at 25°C; 30 min at 55°C; and 5 min at 85°C. The cDNA synthesized was conserved at −20°C until quantitative PCR (qPCR) assays.

### Quantitative PCR and FlaA and FlaB Expression Analysis

After cDNA synthesis, qPCR assays were performed using primers purchased from Eurogentec (Seraing, Belgium; Table 1) and specific for the gene coding for the *flaA* and *flaB* subunit genes. Primers were designed using LightCycler Probe Design Software (version 2.0; Roche Applied Science) or the free online Primer3 software (version 0.4.0) using available sequences retrieved from GenBank (NCBI). Amplifications were carried out on a LightCycler 480 II instrument using LightCycler 480 software (v. 1.5.0) and a LightCycler 480 SYBR green I master kit (Roche Applied Science) according to the provided instructions. The amplification program was as follow: a first hot start (95°C for 10 min) and 50 cycles of an activation step at 95°C for 5 s, an annealing step at 62°C for 5 s, and an elongation step at 72°C for 8 s. Each sample was run in duplicate. A single acquisition of fluorescence for calculation of the *Ct* was processed during the elongation step. The specificity of amplification was verified by size visualization of the PCR product (Table 1) after electrophoresis on a 1.8% agarose gel (Sigma-Aldrich) in 1% TBE (Tris-borate-EDTA) for 30–45 min at 120 V and by analysis of the melting curves of the PCR products (melting temperatures, *T*_m_, in Table 1). All *Ct* values were analyzed using the qbase^PLUS^ software (Biogazelle, Belgium).

**TABLE 1:** Details and sequence of primers used for qPCR assays.

**Gene name**	**Locus tag^a^**	**Sequence (5′-3′)^b^**	***T*_m_ (°C)^c^**	**Size (pb)^d^**
*flaA1*	LIC10788	(F) AGCAAGCGTATCAAGCGA	81.1	151
		(R) GCATTCTCTCCTGGATAAGTG		
*flaA2*	LIC10787	(F) CGTCAGAGGATTTGATAGAGTG	80.3	210
		(R) CCAGGAATTGTAGCGGT		
*flaB1*	LIC11890	(F) GCTGACGGTTCTCTCCTGAC	80.1	280
		(R) ACGTTAGCCTGAGCAAGCAT		
*flaB2*	LIC11889	(F) AGCGAGACAACTTCTTCCGCCATA	78.4	150
		(R) ATGAAGCAGAGAGCGGATATGGGA		
*flaB3*	LIC11532	(F) GCAAGCGCAAACGCTATGAT	79	180
		(R) ATCCCTCACACGGCTTTCTG		
*flaB4*	LIC11531	(F) ACTCCTTACCGGGGCTTTTG	78.8	200
		(R) TCACAGAGTTTGCCTTGCCA		
*lipL21*	LIC10011	(F) TGGTGAAGCTACTGCATCT	80.0	164
		(R) CACCTGGAAATTTTGCG		
*lipL36*	LIC13060	(F) GGTTCAAATTGCGCTGTAG	80.8	188
		(R) GCATAAACGGTTTTTCCGAG		
*lipL41* ^e^		(F) TTTACCAGTTGCCATAGAAGCGGC	77.6	150
		(R) GGAAATCTGATTGGAGCCGAAGCA		

*^*a*^Locus tag of corresponding gene sequence from *L. interrogans* serovar Copenhageni strain Fiocruz L1-130 referenced in GenBank (NCBI) under accession number NC_005823.1 and used for primer design. ^*b*^(F) and (R), forward and reverse primer sequences, respectively. ^*c*^Tm, PCR product melting temperature. ^*d*^PCR product size (in base pairs). ^*e*^As described by Carrillo-Casas et al. (60).*

For *in vivo* infections, the level of expression of each target gene was normalized to the levels of *lipL21*, *lipL36*, and *lipL41* gene, previously validated as reference genes in our conditions (26). The relative normalized expression ratio was then calculated as the ratio of the *in vivo* to the *in vitro* expression level of bacteria cultured at 30°C. For the *in vitro* bacterial cultures, level of expression of *flaB* genes was normalized to the level of *lipL41* housekeeping gene (Normalized relative quantities).

### Generation of Bone Marrow-Derived Macrophages (BMM)

Bone marrow cells (BMC) were obtained as recently described (22). Briefly, mice were euthanized and femurs dissected, cleaned, and the femur heads were cut off. BMC were flushed out using a 21G needle to inject culture medium through the bones. BMC were centrifuged (300 × *g*, 5 min) and treated with Red Blood Cell Lysis Buffer (Sigma-Aldrich) for 10 min, followed by PBS washing. BMC were counted, and 5 × 10^6^ cells seeded in 100 cm^2^ cell culture dishes in 12 mL RPMI supplemented with 10% fetal calf serum (Lonza), 1X non-essential amino acids (NEEA, Gibco), 1 mM sodium pyruvate (complete medium) supplemented with 1X Antibiotic/Anti-mycotic solution (Gibco) and 10% L929 cell supernatant to provide a source of M-CSF1. Cells were incubated for 7-day at 37°C with 5% CO_2_. At day 3, 5 mL of the same medium was added. At day 7, the medium was removed, and 3 mL of cell dissociation buffer (Gibco) was added to harvest the bone marrow macrophages (BMMs). BMMs were collected by scrapping, centrifuged, enumerated and seeded in 96-well plates at a density of 2 × 10^5^ cells per well in complete medium without antibiotics. BMMs were rested for 2–4 h and stimulated for 24 h with different leptospiral strains, live or heat-killed for 30 min at 100°C, at a MOI of 1:100, or 1:50 or with 100 ng/mL of controls [Standard Flagellin from *Salmonella typhimurium* (FLA) and LPS *E. coli* ultra-purified (both from InvivoGen)]. The keratinocyte-derived (KC/CXCL1) was measured in cell supernatants 24 h post-stimulation, by ELISA using Duo-Set kits (R&D Systems), according to the supplier’s instructions.

### TLR5/NF-κB Assay in Human Epithelial Cell Line HEK-Blue-KD-TLR5

Human embryonic kidney (HEK)-Blue-KD-TLR5 cells (Invivogen) are HEK293 cells knocked-down (KD) for TLR5. In these HEK-BLUE cells, the activation of NF-κB drives the expression of the secreated alkaline phosphatase (SEAP) enzyme that induces a color shift from pink to blue of the chromogenic substrate in the HEK-Blue Detection Media (Invivogen). These cells were cultured in complete DMEM medium composed of DMEM GlutaMax (Gibco) with 1 mM sodium pyruvate (Gibco), 1X NEEA (Gibco) and 10% V/V heat-inactivated fetal calf serum (Hi FCS, Gibco). On day 1, cells were detached by 1 min incubation in cell dissociation buffer (Gibco) followed by gentle flush with medium. Cells were then seeded in 22.1 cm^2^ cell culture dishes (TPP) at less than 30% confluence and incubated overnight at 37°C, 5% CO_2_. Cell transfections were performed on day 2, whilst the cells remained under 60% confluence and with a total amount of 3 μg of DNA per dish. For each dish, between 100 ng to 1 μg of pUNO1-humanTLR5, pUNO1-murineTLR5 (Invivogen), pcDNA3.1-bovine TLR5 (27) or the corresponding empty vector was used, complemented up to 3 μg with pcDNA3.1. The transfection reagent 1X FuGENE HD (Promega) in serum free OptiMEM (Gibco) was incubated during 25 min with the DNA followed by transfection of the cells according to the manufacturer’s instruction. On day 3, transfected HEK-Blue-KD-TLR5 cells were stimulated in 96-wells plates. Briefly, 20 μL Flagellin from *Salmonella typhimurium* as a control (Standard FLA-ST (Invivogen) or leptospires resuspended in PBS, at a MOI between 1:50 to 1:200 were added in empty wells. Transfected HEK-Blue-KD-TLR5 cells were then gently flushed in PBS and resuspended in HEK-Blue Detection Media (Invivogen) at 2.8 × 10^5^ cells/mL. 180 μL of cell suspension, corresponding to 50 000 cells, were then added on top of the agonists in each well and plates were incubated for 24h at 37°C, 5% CO_2_. In each well, the activation of NF-κB through TLR5 was assessed by absorbance measurements at 630 nm. All heat treatments were performed under agitation at 300 rpm and in PBS on the diluted leptospires preparations right before addition in the wells. Proteinase K treatments of leptospires (from *Tritirachium album*, Qiagen) were performed under agitation at 300 rpm in PBS for 2 h at 37°C, to avoid killing the leptospires. Such treatment was followed by heat inactivation of the enzyme and bacteria at 100°C for 30 min. The non-inactivated fraction and mock treatment without leptospires were also tested on HEK-Blue-KD-TLR5 cells. Leptospires in PBS were also treated with antimicrobial peptides: LL-37 (InvivoGen) and Bmap28 (Protegenix) at different concentrations (0–250 μg/mL) for 2 h.

### Alamar Blue Viability Assay for Leptospires

Survival of leptospires upon treatments with antimicrobial peptides was assessed by Alamar Blue viability assay (28). After 2 h incubation with LL-37 or Bmap28 in PBS, 2.5 × 10^6^ leptospires in 100 μL in 96-well plates were mixed with 80 μL of EMJH and 20 μL of 10× Alamar Blue dye. Plates were incubated in a humid container at 30°C for 72 h. Viability of leptospires was visible by the color shift from blue to pink upon resazurin reduction to resorufin by live bacteria. Heat-killed (30 min, 100°C) leptospires were used as control for loss of viability.

### *In silico* Analyses of the Flagellin Protein Sequences

All the *in silico* analyses were performed using either Uniprot or GeneBank available sequences. All corresponding accession numbers are mentioned in the figure legends. Amino acid sequence homology percentage (identity) was obtained using BLAST. Alignments of the sequences were performed with MEGA X (29) and using the Clustal method. Structural predictions based on amino acid sequences were obtained using the Phyre2 (30) and figures colored and modified with Chimera (31).

### Statistical Analyses

Statistical analyses were performed using the Student *t-*test, with asterisks corresponding to the following *p* values: ^∗^*p* < 0.05; ^∗∗^*p* < 0.01; ^∗∗∗^*p* < 0.001.

## Results

### TLR5 Deficiency Does Not Modify the Course of Acute Leptospirosis in Mice

**FIGURE 1:**
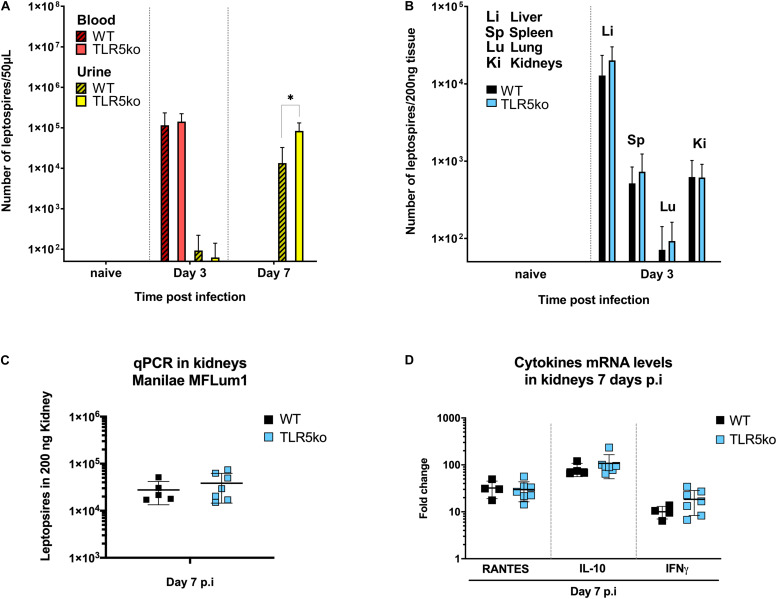
No difference in leptospiral loads and inflammatory mediators between WT and TLR5ko mice during acute phase of leptospirosis. **(A,B)** Bacterial loads determined by qPCR of leptospiral DNA at several days post intraperitoneal infection of 7-week old female C57BL/6J (WT) mice and TLR5ko mice with 10^7^ *L. interrogans* Manilae L495 derivative strain MFLum1; **(A)** in blood (red) and urine (yellow) in WT (*n* = 4, hatched bars) and TLR5ko mice (*n* = 4, empty bars) and **(B)** in liver (Li), spleen (Sp), lungs (Lu) and kidneys (Ki) from WT (*n* = 4, black bars) and TLR5ko (*n* = 4, blue bars) at day 3 post-infection (p.i). Data are expressed as mean (±SD) of all mice. Statistically significant differences between genotypes (Student *t*-test) are indicated. **(C)** Bacterial loads in kidneys determined by qPCR 7 days p.i of WT mice (*n* = 5, black dots) and TLR5ko mice (*n* = 7, blue dots). **(D)** Inflammation measured in kidney by mRNA expression of cytokines (RANTES, IL10, IFNγ) measured by RT-qPCR at 7 days p.i in WT mice (*n* = 5, black dots) and TLR5ko mice (*n* = 7, blue dots). Individual mice are represented as dots and lines correspond to mean (±SD) of all mice. No statistical difference was found between WT and TLR5ko mice. *p < 0.05.

To study the potential involvement of the TLR5 receptor in the host defense against leptospires, we used a murine model of leptospirosis and compared the susceptibility of C57BL6/J (WT) mice *versus tlr5* knock-out (TLR5ko) mice after intraperitoneal infection with a sublethal dose of 10^7^*L. interrogans* [serovar Manilae strain L495 MFlum1 (23)]. We previously showed in this C57BL6/J mouse model that leptospires disseminate and grow in blood until day 3 and from day 4 post-infection (p.i) progressively disappear from the blood circulation and organs (23). At day 7 p.i, pathogenic leptospires are exclusively found in urine, and in small numbers in kidney, where they progressively grow to establish a stable and lifelong renal colonization from 1-month p.i on. At 15 days p.i, leptospires are easily detected in kidneys either by qPCR or by IVIS imaging (23, 32). Here, leptospiral loads were measured by qPCR in blood and urine (Figure 1A) and organs (Figure 1B) in the first week p.i corresponding to the acute phase of infection. As expected, we found leptospires in blood, liver, spleen, lungs and kidney at day 3 p.i (23) (Figures 1A,B), and no difference of leptospiral loads could be observed between WT and TLR5ko mice in blood and organs. At day 7 p.i, leptospires were detected in urines but not in blood (Figure 1A), similar as previously observed (23). However, at day 7 p.i, we measured more leptospires in the urine of TLR5ko mice than in WT mice. Nevertheless, at day 7 p.i, we did not observe any difference in bacterial loads in kidneys between WT and TLR5ko mice (Figure 1C). In addition, mRNA expression of pro-(IFNγ), anti-(IL-10) inflammatory cytokines and RANTES chemokine measured by RT-qPCR at day 7 p.i in the kidneys did not differ between WT and TLR5ko mice (Figure 1D). Altogether these results suggest that the presence of TLR5 does not play a major role in the murine defense at the acute phase of experimental leptospirosis.

### TLR5 Deficiency Does Not Impact Renal Colonization

**FIGURE 2:**
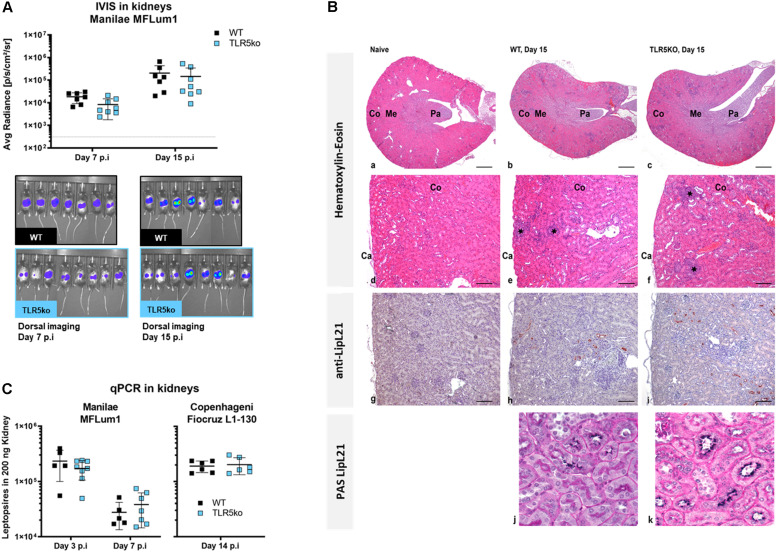
No difference in kidney colonization in WT and TLR5ko mice 15 days post-infection. **(A)** Bacterial loads in urine quantified by qPCR (left panel), and quantification and images of live imaging (IVIS) (right panel) 15 days post-infection (p.i) of 7-week old female WT mice (*n* = 7, black) and TLR5ko mice (*n* = 8, blue) with 10^7^ *L. interrogans* Manilae bioluminescent derivative strain MFLum1. Imaging was performed in dorsal position, 24 h post shaving, on anesthetized mice and after luciferin administration. The background level of light after luciferin administration was measured on a control TLR5ko mouse injected with PBS at the time of infection (dotted line). The average radiance in individual mice gated on the whole body is shown and represented as dots; lines correspond to the mean (±SD) of all mice. No statistical difference was found between WT and TLR5ko mice. **(B)** Bacterial loads in kidneys determined by qPCR of leptospiral DNA 14 days p.i of WT mice (*n* = 6, back dots) and TLR5ko (*n* = 6, blue dots) mice with 10^7^*L. interrogans* Copenhageni Fiocruz L1-130. Individual mice are shown and represented as dots; lines correspond to mean (±SD) of all mice. No statistical difference was found between WT and TLR5ko mice. **(C)** Histological sections and immunolabeling of the kidneys of naive TLR5ko, infected WT and TLR5ko mice 15 days p.i with 10^7^ *L. interrogans* Manilae strain MFLum1. **(a–c)** Kidney, Hematoxylin-Eosin stain, Original magnification ×2, Scale bar: 500 μm. Cortex (Co), Medulla (Me), Papilla (Pa), Capsule (Ca). **(d–f)** Kidney cortex, Hematoxylin-Eosin stain, Original magnification ×10, Scale bar: 100 μm. The asterisks indicate the focal inflammatory infiltrates. **(g–i)** Anti-LipL21 labeling of leptospires in renal tubules, Original magnification ×10, Scale bar: 100 μm. **(j,k)** Double labeling LipL21/Periodic Acid-Schiff (PAS) to stain the PAS positive brush borders present in proximal tubules only. Original magnification ×40, Scale bar: 25 μm.

Next, we established whether the presence of TLR5 is a necessary pre-requisite for the establishment of colonization or the localization of leptospires in the kidneys (18, 32). In contrast to day 7 p.i, we found equivalent loads of leptospires in urine from both genotypes 15 days p.i (Figure 2A). Next, we used the bioluminescent property of *L. interrogans* Manilae strain MFLum1 to quantify and visualize leptospires by IVIS imaging in the kidneys of WT versus TLR5ko mice 15 days p.i. The levels and shape of emitted light, reflecting live bacteria (23), were equivalent between WT and TLR5ko infected mice (Figure 2B). In addition, we infected mice with 10^7^*L. interrogans* Copenhageni Fiocruz strain L1-130, another serovar of pathogenic *L. interrogans*, and also found by qPCR 15 days p.i equivalent leptospiral loads in kidneys of WT and TLR5ko mice (Figure 2C). Using immunohistochemistry, we further investigated the presence and localization of Manilae L495 leptospires in kidneys of WT and TLR5ko mice, as well as the *Leptospira*-induced nephritis 15 days p.i (18). Minimal inflammatory cellular infiltrates were noted in the cortex of both WT and TLR5ko infected mice (Figure 2C a–f), whereas no inflammation was observed in the naive WT control. Labeling of leptospires with an anti-LipL21 antibody (17) revealed *Leptospira*-infected tubules in the renal cortex, as already described (18) (Figure 2C g–i). In histological sections of the kidney stained with Periodic Acid-Schiff (PAS), we found only leptospires in some proximal tubules in both WT and TLR5ko mice associated with the PAS positive microvilli of the brush border at the luminal surface of the tubular epithelium (Figure 2C j,k), as previously described in rats (33). Altogether, the quantitative assessment by IVIS and qPCR, in combination with the qualitative results obtained by immunohistochemistry suggest that TLR5 does not play a major role in host protection during the acute or chronic phase of a *L. interrogans* infection in mice.

### Live Pathogenic Leptospires Do Not Signal Through Murine and Human TLR5 *in vitro*

**FIGURE 3:**
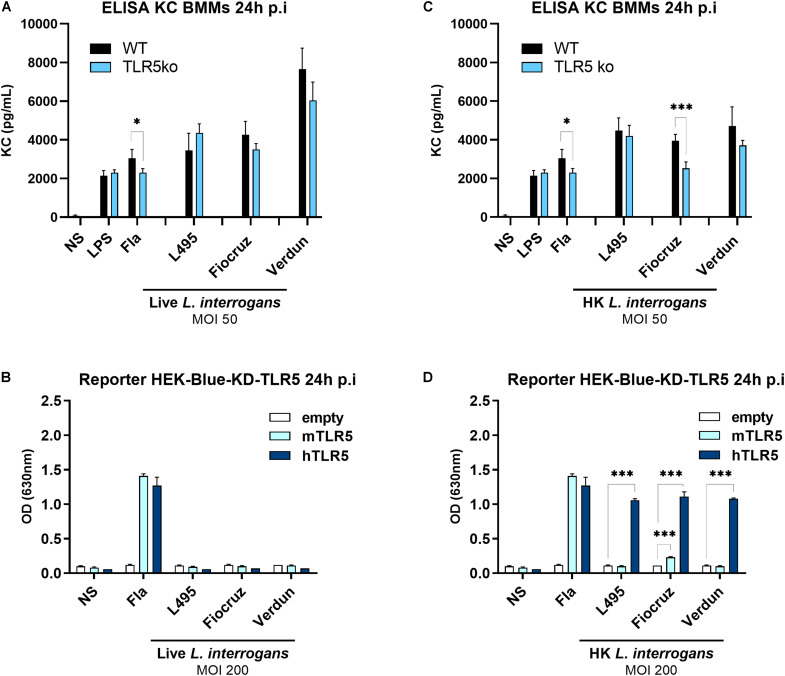
Heat-killed, but not live leptospires, signal through hTLR5. **(A,C)** KC production measured by ELISA in the supernatants of BMMs from WT (black bars) and TLR5ko (blue bars) mice 24 h post-infection with MOI 50 of either **(A)** live or **(C)** heat-killed (30 min, 100°C) different serovars of virulent *L. interrogans* (Manilae strain L495, Copenhageni strain Fiocruz L1-130, Icterohaemorrhagiae strain Verdun). LPS from *E. coli* (100 ng/mL) and unpurified Fla from *Salmonella typhimurium* (500 ng/mL) were used as controls. Data are expressed as mean (±SD) of technical replicates (*n* = 5) on pooled BMMs preparations from mice (*n* = 3) and are representative of at least three independent experiments. Statistically significant differences between genotypes (Student *t*-test) are indicated. **(B,D)** NF-κB reporter assay in HEK-Blue-Knock Down (KD)-TLR5 cells transfected with the mouse TLR5 (light blue bars), human TLR5 (blue bars), or empty plasmid (empty bars) and stimulated for 24 h with MOI 200 of either **(B)** live or **(D)** heat-killed (30 min, 100°C) different serovars of virulent *L. interrogans*. Unpurified Fla from *Salmonella typhimurium* (100 ng/mL) was used as control. Data are expressed as the mean (±SD) of technical replicates (*n* = 3) and are representative of at least three independent experiments. Statistically significant differences (Student *t*-test) are indicated. *p < 0.05; ***p < 0.001.

To further investigate the role of TLR5 in recognition of *Leptospira*, bone marrow derived macrophages (BMMs) from WT and TLR5ko mice were infected with 3 different live serovars of *L. interrogans.* The production of the murine chemokine KC (CXCL1) was measured by ELISA 24 h p.i in the cellular supernatants. This chemokine was chosen as it was recently shown to be fully dependent on the MyD88-dependent signaling pathway (34), with MyD88 being the first main adaptor involved in TLR5-induced signaling (35). We therefore considered changes in KC secretion to be a good readout for analyzing TLR5 contribution to the leptospiral-induced signaling. We did not find any difference between both genotypes (Figure 3A), which correlated with the *in vivo* experiments and indeed strongly supports the observation that live leptospires do not induce signaling through murine TLR5.

Next, we assessed whether the lack of signaling through or escape of recognition by murine TLR5 is a species-specific phenomenon. Indeed, we previously highlighted PRR species-specificities of leptospiral MAMPs recognition, such as murine TLR4 receptor that only partially recognizes the leptospiral LPS (34), whereas human TLR4 does not. Conversely, human, but not the murine NOD1 is able to sense leptospiral muropeptides (17, 36). We therefore used the human HEK-blue reporter cells, with a NF-κB promoter driving SEAP expression, which can be measured using a colorimetric test. We transfected HEK-Blue-KD-TLR5 cells, which are Knocked-Down for TLR5, with either human TLR5, murine TLR5, or an empty control vector. No signal corresponding to murine or human TLR5-mediated NF-κB activation was obtained upon infection with different live *L. interrogans* serovars at MOI 10 and 100 (data not shown) or even at an MOI of 200 (Figure 3B), which suggested that leptospires also evade human TLR5 recognition or at least do not signal through this TLR.

### Heat-Killed Leptospires Signal Through Human TLR5, but Only the Heat-Killed Fiocruz Strain Signals Through Murine TLR5

The specificity of TLR5 activation and resulting signaling is usually assessed by denaturing a potential ligand through heat-inactivation. In the present study, we observed that inactivation of strains Manilae L495 and Icterohaemorrhagiae Verdun at 100°C for 30 min induced equivalent levels of KC production in WT and TLR5ko murine BMMs (Figure 3C), which was consistent with the results obtained using live bacteria (Figure 3A). In contrast, the heat-killed Copenhageni Fiocruz strain L1-130 induced less KC secretion in TLR5ko compared to WT BMMs (Figure 3C), suggesting that an agonist present in the inactivated Fiocruz strain could be recognized by murine TLR5. Unexpectedly, heat-killed leptospires from all serovars strongly activated HEK-Blue-KD-TLR5 transfected with human TLR5 (Figure 3D). Further, despite the fact that both strains, Manilae L495 and Icterohaemorrhagiae Verdun, did not stimulate murine TLR5, a slight activation signal was observed with the Copenhageni Fiocruz L130 strain, which was consistent with the chemokine result in BMMs (Figure 3C). The experiment was performed in parallel with an empty plasmid, showing that these results were indeed specific to TLR5 activation, and did not depend on a different NF-κB activation pathway (Figure 3D). Altogether these unexpected results suggested that only heat-killed leptospires can signal through human TLR5, but not or only barely through murine TLR5, providing a new example of species-specificity of PRR recognition of leptospiral MAMPs.

### A Heat-Resistant Protein From Heat-Killed Leptospires Signals Through TLR5

**FIGURE 4:**
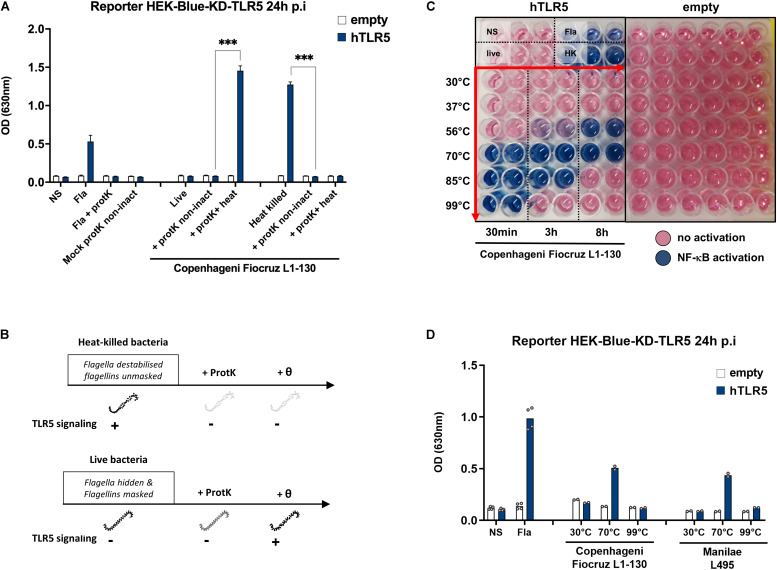
A very stable protein from leptospires signal through TLR5. **(A)** NF-κB reporter assay in HEK-Blue-KD-TLR5 cells transfected with the human TLR5 (blue bars), or empty plasmid (empty bars) and stimulated with MOI 100 of either live or heat-killed (30 min, 100°C) *L. interrogans* Copenhageni strain Fiocruz L1-130 treated or not with Proteinase K (protK) followed or not by heat inactivation at 99°C for 30 min (inact or non-inact). Unpurified Fla from *Salmonella typhimurium* (100 ng/mL) was used as control. Data are expressed as the mean (±SD) of technical replicates (*n* = 3) and are representative of at least three independent experiments. Statistically significant differences (Student *t*-test) are indicated. **(B)** Chronogram of proteinase K experiments. **(C)** Picture of NF-κB reporter assay in HEK-Blue-KD-TLR5 cells transfected with the human TLR5 or empty plasmid and stimulated with MOI 100 of live *L. interrogans* Copenhageni strain Fiocruz L1-130 incubated at various temperatures during 30 min, 3, or 8 h. Unpurified Fla from *Salmonella typhimurium* (500 ng/mL) was used as control. Picture show technical duplicate for each condition and is representative of at least three independent experiments. **(D)** NF-κB reporter assay in HEK-Blue-KD-TLR5 cells transfected with the human TLR5 (blue bars) or empty plasmid (empty bars) and stimulated with MOI 100 of live *L. interrogans* Copenhageni strain Fiocruz L1-130 or Manilae L495 incubated at various temperatures during 3 h. Unpurified Fla from *Salmonella typhimurium* (500 ng/mL) was used as control. Data are expressed as the mean of technical replicates (*n* = 2, shown as dots) and are representative of at least three independent experiments for Fiocruz L1-130 and two independent experiments for L495. ***p < 0.001.

To our knowledge, our results showing TLR5 activation using heat-inactivated leptospires have never been described before. Thus, we first ensured that the signal observed was indeed attributed to proteins of leptospires interacting with TLR5. Since only the stimulation with heat-killed bacteria resulted in TLR5 signaling, we anticipated that a proteinase K treatment would destroy the protein involved in the signaling. Therefore, we treated live and heat-killed Fiocruz L1-130 leptospires with proteinase K, followed or not by heating at 100°C for 30 min to inactivate the enzyme. We stimulated TLR5 transfected HEK-Blue-KD-TLR5 with these preparations and a mock control without bacteria. Although the proteinase K treatment had no effect on live bacteria, it decreased the signal on heat-killed bacteria (Figure 4A). In contrast to live bacteria treated with proteinase K and subsequently heated, which resulted in a strong TLR5 activation, TLR5 signaling was not restored in heat-killed bacteria treated with proteinase K, suggesting that the proteinase K had digested all TLR5 agonists (Figure 4A). This experiment confirmed the protein nature of the agonist present in heat-killed leptospires, which was not affected by proteinase K digestion in live bacteria. We hypothesize that in live leptospires, the periplasmic location of the endoflagella could protect the flagellin subunits from proteinase K digestion, thus potentially explaining why live bacteria do not signal through TLR5 and are not affected by the enzyme (Figure 4B).

Next, we investigated the unusual thermostability of the TLR5 agonist, by incubating live Fiocruz L1-130 leptospires at different temperatures (from 30°C and up to 99°C) and for different durations (from 30 min and up to 8 h) (Figure 4C) before stimulation of HEK-Blue-KD-TLR5 transfected with human TLR5. Interestingly, after 8 h incubation at 30°C (the optimal temperature for leptospiral growth *in vitro*) or at 37°C (the host temperature), we did not observe any TLR5-dependent signaling. Of note, at 56°C, the usual temperature to inactivate leptospires (15, 18) whilst keeping the leptospiral shape integrity, a signal started after 3 h of incubation, but even 8 h were not enough to get a full TLR5 signaling. At 70°C, the temperature classically used to depolymerize the *Salmonella*’s flagellum filament (10), a 30 min incubation was sufficient to stably activate TLR5 for 8 h. The signal observed with leptospires incubated for 30 min at 85°C disappeared after 8 h, whereas the positive signal observed after heating the bacteria at 99°C for 30 min disappeared after 3 h of heating (Figure 4C). We then tested in parallel the Manilae L495 and Copenhageni Fiocruz L1-130 strains, after heating at 30, 70, and 99°C for 3 h, and obtained similar results for both 2 serovars (Figure 4D). These results confirmed the protein nature of the TLR5 agonist of leptospires, since the activation can be extinguished by heating the bacteria for an extended time at high temperature.

### Antimicrobial Peptides Destabilize Live Leptospires and Unmask a TLR5 Signal

**FIGURE 5:**
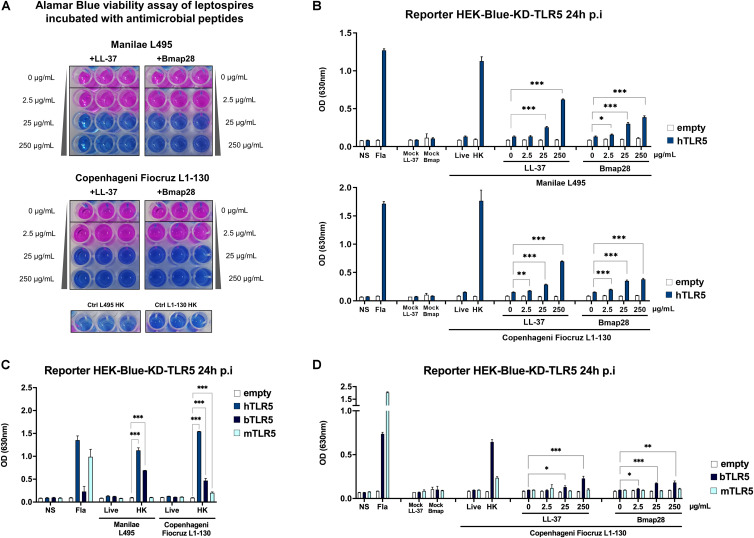
Human and bovine antimicrobial peptides unmask the leptospiral ability to signal through human and bovine TLR5 receptors. **(A)** Alamar blue viability assay of leptospires (Manilae L495 or Copenhageni Fiocruz L1-130) incubated with increasing concentration (0–250 μg/mL) of antimicrobial peptides LL-37 or Bmap28 for 2 h. Heat-killed (30 min, 100°C) leptospires are used as controls for loss of viability. Picture show technical triplicate for each condition and is representative of two independent experiments. **(B)** NF-κB reporter assay in HEK-Blue-KD-TLR5 cells transfected with the human TLR5 (blue bars), or empty plasmid (empty bars) and stimulated with MOI 100 of *L. interrogans* Manilae strain L495 or Copenhageni strain Fiocruz L1-130 treated with human peptide LL-37 or bovine peptide Bmap28 at various concentration (0–250 μg/mL) for 2 h before stimulation. **(C)** NF-κB reporter assay in HEK-Blue-KD-TLR5 cells transfected with the human TLR5 (blue bars), bovine TLR5 (dark blue bars), mouse TLR5 (light blue bars) or empty plasmid (empty bars) and stimulated with MOI 100 of either live or heat-killed (30 min, 100°C) *L. interrogans* Manilae strain L495 or Copenhageni strain Fiocruz L1-130. **(D)** NF-κB reporter assay in HEK-Blue-KD-TLR5 cells transfected with the bovine TLR5 (dark blue bars), mouse TLR5 (light blue bars) or empty plasmid (empty bars) and stimulated with MOI 100 of *L. interrogans* Copenhageni Fiocruz L1-130 treated with human peptide LL-37 or bovine peptide Bmap28 at various concentration (0–250 μg/mL) for 2 h before stimulation. **(B–D)** Unpurified Fla from *Salmonella typhimurium* (500 ng/mL) was used as control. Data are expressed as the mean (±SD) of technical replicates (*n* = 3), and are representative of at least three independent experiments for panels **(A)** and **(C)**. Statistically significant differences (Student *t*-test) are indicated. *p < 0.05; **p < 0.01; ***p < 0.001.

Since we revealed the potential for TLR5 recognition of leptospires by heating at high non-physiological temperatures, we wondered whether leptospires could signal through TLR5 after being destabilized or killed with antibiotics or antimicrobial peptides. Antibiotic treatments of the leptospires (at MIC concentrations) including gentamicin, azithromycin, daptomycin and penicillin G, the latter being known to target the cell wall, didn’t induce any TLR5 signal (Supplementary Figure 1A). Next, we tested the effect of two different antimicrobial peptides (AMP), LL37 and Bmap28. Cathelicidin LL-37 is an AMP which has been shown to be active against leptospires (37), and its presence was recently associated with a better outcome in human patients with leptospirosis (38). Furthermore, LL-37 has also been shown to prevent death in young hamsters experimentally infected with the Fiocruz L1-130 strain (38). The second AMP, bovine Bmap28 has been described to be 50–100 times more efficient in killing leptospires compared to LL-37, but this depended on the serotypes (37). Therefore, we first tested the ability of both AMP to kill strains Manilae L495 and Copenhageni Fiocruz L1-130. Using an Alamar blue viability assay, we observed that both strains were killed after 2 h of incubation with 25 μg/mL of either LL-37 or Bmap28 (Figure 5A). Next, we assessed whether leptospires treated with the different doses of AMP were recognized by human TLR5. Interestingly, both, live *L. interrogans* Manilae L495 and Copenhageni Fiocruz L1-130 bacteria pre-treated with either 25 or 250 μg/mL of LL-37 or Bmap28 induced a significant and dose-dependent signal in human TLR5 (Figure 5B). These results suggest that antimicrobial peptides could participate *in vivo* in the exposure of flagellins and subsequently recognition by and signaling through TLR5.

As one of the AMP used was of bovine origin (bovine Bmap28) and pre-treatment of leptospires with Bmap28 had a clear effect on human TLR5 signaling, we tested whether bovine TLR5 could indeed recognize leptospires. In accordance with published data (27), bovine TLR5 reacted only weakly to the positive control, Salmonella derived FliC (Figure 5C). In contrast, we found that it recognized heat-killed Manilae L495 and Copenhageni Fiocruz L1-130 (Figure 5C). Interestingly, the treatment of live Fiocruz L1-130 (Figure 5D) and live L495 (Supplementary Figure 1B) with both AMP (LL37 and Bmap28) resulted in a dose-dependent bovine TLR5 signaling response. However, both treatments, even at high concentration, did not result in a significantly increased signaling response when leptospires were incubated on cells expressing murine TLR5 (Figure 5D and Supplementary Figure 1B). These data suggest that, *in vivo*, degraded leptospires could be recognized by and signal through human and bovine TLR5, and confirm that mouse TLR5 does not recognize leptospires (Figure 5D).

### *In silico* Analyses of Potential TLR5 Binding of Leptospiral FlaBs

**FIGURE 6:**
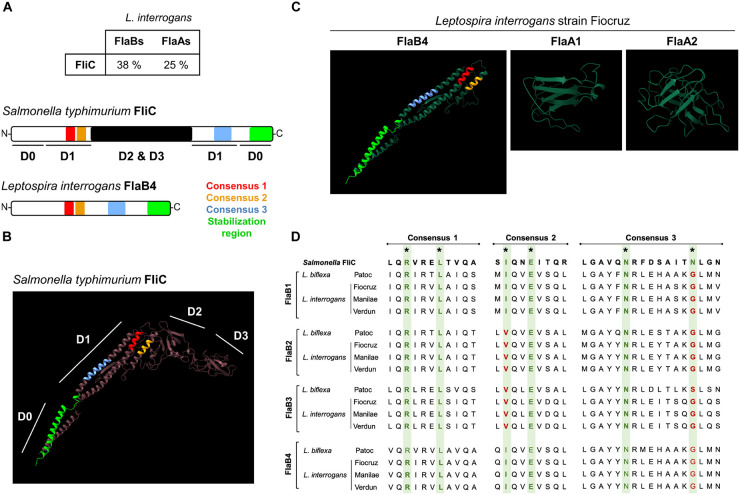
Comparison of leptospiral Flagellins and FliC structures in relation with TLR5. **(A)** Amino acid sequence homology average percentage between *Salmonella typhimurium* FliC (P06179) and *Leptospira interrogans* strain Fiocruz FlaB (LIC11890, LIC11889, LIC11532 and LIC11531) and FlaAs (LIC10788 and LIC10787) and primary structures of the flagellin proteins with TLR5 binding consensus. **(B)** *In silico* (Phyre2 and Chimera softwares) prediction of *Salmonella typhimurium* FliC (P06179) structure with the four described domains and with positions of the TLR5 binding consensus: 1 (red), 2 (yellow) and 3 (light blue) and stabilization region (light green) highlighted. **(C)** *In silico* (Phyre2 and Chimera softwares) prediction of *Leptospira interrogans* strain Fiocruz FlaB4 (LICI1531) with the positions of the TLRS binding consensus and stabilization region highlighted, FlaA1 (LIC10788), FlaA2 (LICI0787). **(D)** Clustal (MEGA software) alignment of the amino acid sequences for the TLR5 binding consensus regions of: *Salmonella enterica* FliC (GeneBank QDQ31983.1), *L. bifleva* (strain Patoc) FlaB1 (LEPBla2133), FlaB2 (LEPBIa2132), FlaB3 (LEPBla1872) and FlaB4 (LEPBla1589), *L. interrogans* (strain Fiocruz L1-130) FlaB1 (LIC18890), FlaB2 (LICIT889), FlaB3 (LICI1532) and FlaB4 (LIC11531), *L. interrogans* (strain L495) FlaB1 (LMANv2 260016), FlaB2 (LMANv2 260015). FlaB3 (LMANv2 590024) and FlaB4 (LMANv2 590023), and *L. interrogans* (strain Verdun) FlaB1 (AKWP_v1_110429), FlaB2 (AKWP_v1_110428) and FlaB3 (AKWP_v1_110068) and FlaB4 (AKWP_v1_110067).

Two leptospiral *flaA* (*flaA1* and *flaA2*) genes and four *flaB* genes (*flaB1* to *flaB4*) have been annotated in the *L. interrogans* genomes according to their similarity with the *Salmonella* flagellin (FliC), the two families sharing respectively around 25 and 38% identity at the protein level with FliC (Figure 6A). Structural studies recently showed that the FlaB subunits constitute the core of the flagellum, and the other subunits constitute an asymmetric outer sheath, with FlaB interacting with FlaA on the concave site and with FcpA on the other side of the curvature. FcpA and FcpB associate in a lattice forming the convex part of the endoflagellum (8) (Supplementary Figure 2A). Using the BLAST-P software, we found that the different FlaB subunits from the *L. interrogans* Fiocruz strain share 57–72% of identity and most probably result from gene duplication events (Supplementary Figure 3A). Similar results were obtained with the saprophytic *L. biflexa* Patoc strain (Supplementary Figure 3A). We then used the Phyre2 software to model the FlaBs structures according to their primary amino acid sequences, using the FliC protein sequence as a base. FliC folds in four regions D0 to D3, forming an inverted L shape (Figure 6B), with both N-term and C-term in the D0 domain. Region D1, in the inner face of the monomer, is involved in the interaction of FliC with the leucine–rich repeat (LRR) domains of TLR5 via 3 binding sites (Figures 6A,B and Supplementary Figure 2B) (39, 40). There is also a region in the C-term part of the D0 domain that is not directly involved in the binding to TLR5 but important for the stabilization of the TLR5 dimers upon binding to FliC (Figures 6A,B and Supplementary Figure 2B) (41). Phyre 2 predictions showed that all FlaB subunits from *L. interrogans* and *L. biflexa* harbor orthologs of the D0 and D1 domains of FliC, while missing the D2 and D3 domains (Figure 6C, and data not shown). We also checked whether FlaA1 or FlaA2 could have a structure mimicking the D2-D3 domains of FliC, but leptospiral FlaA1 and FlaA2 looked globular, mainly presenting ß sheets and do not resemble the missing domains (Figure 6C). Interestingly, we found that the FlaB possessed the 3 conserved sequences important for TLR5 binding in the D1 domain (Figures 6A–D). Then, we compared the different pathogenic *L. interrogans* and the saprophytic *L. biflexa* Patoc I strain and found that the four FlaB, although distinct from each other (Supplementary Figure 3A), were highly conserved in the consensus regions of the TLR5 binding domains in D1 (99–100% identity among the different pathogenic serovars, the Patoc FlaB being less conserved) (Figure 6D). We also found in FlaB the consensus in the D0 domain involved in the flagellin/TLR5 complex stabilization (Figures 6A,C). We compared the leptospiral FlaB sequences in these 3 consensus binding TLR5 regions with other spirochetes, *Borrelia burgdorferi* and *Treponema* spp., the latter known to signal via TLR5 when FlaB are expressed as recombinant proteins (42) and also with bacteria known to dodge the TLR5 response such as *Helicobacter pylori* (43) and *Bartonella bacilliformis* (10), presenting variations in those consensus sequences of their flagellins (Supplementary Figure 4A). In addition, we also found this FlaB region to be 100% conserved in a panel of major species of *Leptospira* circulating all over the world, including potential human pathogens, such as *L. borgpeterseni, L. kirschneri*, *L. noguchii*, *L. weilii*, *L. santarosai*, as well as *L. licerasiae*, belonging to another clade of species of lower virulence (2) (Supplementary Figure 4B). These alignments show that the TLR5 binding site region is highly conserved in all leptospiral FlaBs. Therefore each of the four FlaB subunit could potentially signal through TLR5, since leptospiral FlaBs share the 2 first consensus with TLR5 activating bacteria and the different residue observed in the consensus number 3 is also present in TLR5-activating *Treponema* flagellins (44).

### FlaB, Not FlaA Nor Fcp, Induce TLR5 Signaling

**FIGURE 7:**
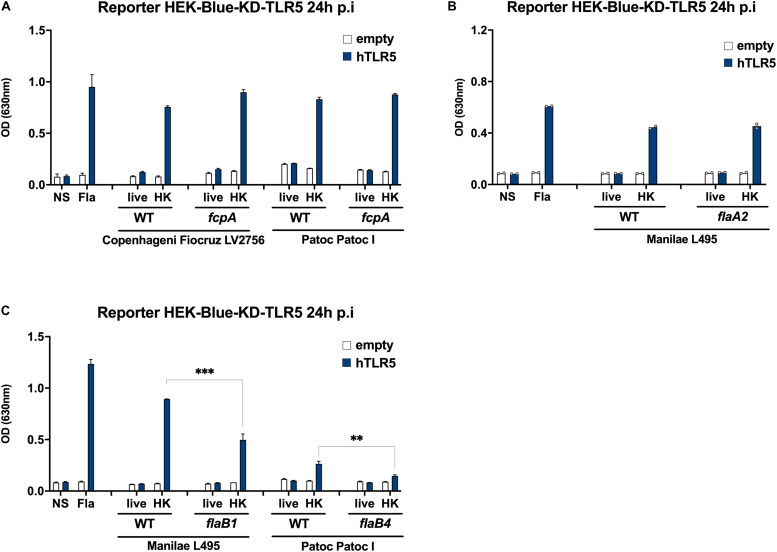
FlaB subunits, not FlaAs nor Fcps, contribute to the signaling. **(A)** NF-κB reporter assay in HEK-Blue-KD-TLR5 cells transfected with the human TLR5 (blue bars), or empty plasmid (empty bars) and stimulated with MOI 100 of either live or heat-killed (30 min, 100°C) *L. interrogans* Copenhageni Fiocruz LV2756 WT or ΔFcpA, Patoc Patoc I WT or ΔFcpA. Unpurified Fla from *Salmonella typhimurium* (500 ng/mL) was used as control. Data are expressed as the mean (±SD) of technical replicates (*n* = 3) and are representative of at least three independent experiments. **(B)** NF-κB reporter assay in HEK-Blue-KD-TLR5 cells transfected with the human TLR5 (blue bars), or empty plasmid (empty bars) and stimulated with MOI 100 of either live or heat-killed (30 min, 100°C) *L. interrogans* Manilae L495 WT or ΔFlaA2. Unpurified Fla from *Salmonella typhimurium* (500 ng/mL) was used as control. Data are expressed as the mean of technical duplicates (*n* = 2, shown as dots). **(C)** NF-κB reporter assay in HEK-Blue-KD-TLR5 cells transfected with the human TLR5 (blue bars), or empty plasmid (empty bars) and stimulated with MOI 100 of either live or heat-killed (30 min, 100°C) *L. interrogans* Manilae L495 WT or ΔFlaB1 and Patoc Patoc I WT or ΔFlaB4. Data are expressed as the mean (±SD) of technical replicates (*n* = 3) and are representative of at least three independent experiments. Statistically significant differences (Student *t*-test) are indicated. **p < 0.01; ***p < 0.001.

To confirm the putative role of the FlaB subunits in inducing TLR5 signaling, we used different available mutants deficient in either FlaA, FlaB or Fcp subunits to stimulate HEK-blue reporter cells transfected with human TLR5. Of note, both *flaAs* and *f* genes are in operons, and the *flaA2* mutant lacks both FlaA1 and FlaA2 subunits (3). Likewise, the *fcpA* mutant lacks both FcpA and FcpB subunits (5, 6). Our results showed that the TLR5 signaling induced with the heat-killed *fcpA* mutants in Fiocruz LV2756 was equivalent to the activation observed with parental strains (Figure 7A). Moreover, we confirmed this result by using a *fcpA* mutant of the saprophytic *L. biflexa* Patoc Patoc I strain (Figure 7A). Likewise, TLR5 signaling was not changed comparing heat-killed WT Manilae L495 and the *flaA2* mutant (Figure 7B). However, the heat-killed *flaB1* mutant induced a lower activation than its parental counterpart (Figure 7C). We also observed a decrease of the TLR5 response with the Patoc I *flaB4* mutant (Figure 7C). These results suggest that the FlaB subunits, but not the FlaA or Fcp, are involved in the TLR5 signaling, and are in line with sequence comparison data.

### FlaB mRNA Are Upregulated in Stationary Phase

**FIGURE 8:**
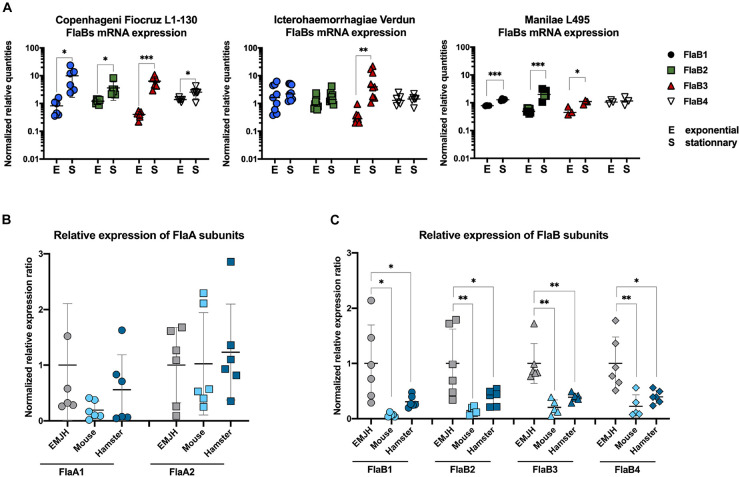
FlaBs mRNA are upregulated in stationary phase and downregulated *in vivo*. **(A)** *In vitro* FlaBs mRNA expression in *L. interrogans* Copenhageni Fiocruz L1-130, Icterohaemorrhagiae Verdun and Manilae L495 at the exponential (E) and stationary (S) phase. Data of RT-qPCR are expressed as the relative mRNA quantities normalized to the expression of the lipl41 mRNA. Technical replicates are represented as dots and lines correspond to mean (±SD) of replicates (3 < *n* < 9). Statistically significant differences (Student *t*-test) are indicated. **(B)** *In vivo* FlaAs and **(C)** FlaBs mRNA expression in blood of infected mice (*n* = 5, light blue) and hamsters (*n*-5, dark blue), 24 h post intraperitoneal infection with 2 × 10^8^ virulent *L. interrogans* Icterohaemorrhagiae strain Verdun, compared with mRNA expression in culture in EMJH at 30°C. Data of RT-gPCR are expressed as the ratio of mRNA quantities relatives to the EMJH control. Individual animals are represented as dots and lines correspond to mean (±SD) of all animals. Statistically significant differences (Student *t*-test) are indicated with corresponding *p* values: * for *p* < 0.05; ** for *p* < 0.01 and *** for *p* < 0.001.

Proteomic and high throughput mass spectrometry performed with the Fiocruz L1-130 strain grown in EMJH have shown that all four *flaBs* genes were expressed and part of the leptospiral flagellum (7). To test whether leptospires could differently regulate the FlaBs expression, cultures of leptospires were harvested after 3–6 days, or after 10–14 days of culture corresponding to exponential growth or stationary phase, respectively. mRNA was purified and RT-qPCR performed with specific primers of the four leptospiral *flaB* genes. The results suggested that the mRNA expression of the different FlaB subunits might vary during bacterial growth *in vitro* (Figure 8A). Indeed, the gene expressions of all FlaB subunits of the Copenhageni Fiocruz L1-130 strain were upregulated at the stationary phase compared to the exponential phase (Figure 8A). In contrast, in the Icterohaemorrhagiae Verdun strain, only the expression of FlaB3 was upregulated at the stationary phase, whereas both, FlaB2 and FlaB3 were upregulated at the stationary phase in the Manilae L495 strain (Figure 8A). Since in prokaryotes the process from transcription to translation is very rapid, these results of *flaB* mRNA expression together with TLR5 sensing suggest an unanticipated upregulation of the FlaB subunits at the stationary phase or conversely a downregulation at the exponential phase that could potentially influence the TLR5 sensing.

### *In vivo* Infection of Rodent Models Leads to Downregulation of *flaB* mRNA

To further investigate whether FlaB regulation could be relevant or play a role *in vivo*, mice and hamsters were infected with the virulent Icterohaemorrhagiae Verdun strain, as described previously (21). Blood was sampled to purify total mRNAs 24 h p.i, when leptospires start their exponential growth in blood of mice (23). In parallel, *in vitro* cultures were performed in EMJH either at 37°C, the host temperature or at 30°C, the usual leptospiral growth culture conditions. First, the expressions of *flaA1* and *flaA2* were not different at 30°C and 37°C (Supplementary Figure 5), nor between the *in vitro* conditions and *in vivo* conditions, either in mice or hamsters (Figure 8B). However, the FlaB expressions were strikingly different, with a weaker expression of the FlaB subunits in the hosts compared to the *in vitro* cultures at 30°C (Figure 8C) or 37°C (Supplementary Figure 5). These data strongly suggest that 24 h p.i, compared to *in vitro* cultures, leptospires downregulate the expression of their FlaBs subunits in animal blood, which as a consequence could participate in the TLR5 avoidance.

## Discussion

In the present study, we showed that live leptospires largely evade induction of signaling through TLR5 and even may escape recognition by TLR5. However, TLR5 agonists were unexpectedly released after boiling for 30 min, and we further showed that these have an unusual thermoresistance. We determined that the TLR5 activity relied, as expected, on the FlaB subunits, known to form the core of the flagella. This subunit also shares some structural features and consensus domains of TLR5 binding with the FliC flagellin subunit of *Salmonella*. Our results also highlight the species specificity of the TLR5 recognition of the leptospiral FlaBs, and potentially differences among serovars. Indeed, we evidenced that human and bovine TLR5 recognized heat-killed leptospires, although the mouse TLR5 did not sense the Icterohaemorrhagiae Verdun and Manilae L495 strains, but recognized the Copenhageni Fiocruz L1-130 strain, although scantily. We showed that antimicrobial peptides were active against live bacteria and allowed for their signaling through human and bovine TLR5, but not through mouse TLR5. Finally, we showed that leptospires downregulated the FlaBs gene expression in blood from both resistant mice and susceptible hamsters, suggesting a mechanism of immune evasion.

Our results of the *in vivo* analyses performed in blood and in organs suggest that TLR5 does not play a central role in the control of leptospires, neither during the acute nor during the chronic phase of infection in a murine model. Whereas, we found a higher number of leptospires in the urine of TLR5ko mice 7 days p.i, we did not find such differences in either urine or kidneys of mice infected with the two different strains, Manilae L495 and Copenhageni Fiocruz L1-130 at 15 days p.i. This paradox is difficult to explain. Although the number of leptospires was normalized by the volume of urine, one parameter that we did not control was the flux of emitted urine that could have biased our result. Further studies with more animals would be required to check that the urine metabolism is not altered in TLR5ko, leading to an apparent higher excretion compared to WT mice.

The absence of TLR5 response in the mouse model was surprising because (i) it was shown that neutralizing TLR5 antibodies decreased the cytokine response of whole human blood upon infection with *L. interrogans* (45), (ii) we showed here that antimicrobial peptides could degrade live leptospires and induce human and bovine TLR5 recognition, and (iii) we previously demonstrated that leptospires were killed and cleared from blood during the first days following infection in mice (23), suggesting the release of free flagellin subunits that could have stimulated the TLR5 response. Hence, our study highlights a species-specificity of the TLR5 recognition since murine TLR5, unlike human and bovine TLR5, was unable to detect the Manilae L495 and Icterohaemorrhagiae Verdun strains. This was unexpected since mouse TLR5 is usually more flexible and able to accommodate more different agonist structures compared to human TLR5 (46), similar to what is seen for human and mouse TLR4 (36). However, the heat-killed Copenhageni Fiocruz L1-130 strain was recognized by mouse TLR5, although to a lesser extent than compared to human TLR5. The weak response seen in mouse TLR5 activation is consistent with our previous study showing equivalent levels of IL1ß release in BMMs from WT and TLR5ko mice infected with live Fiocruz L1-130 strain, although stimulation with heat-killed leptospires triggered less IL1ß in TLR5ko BMMs (15). Interestingly, we previously showed by microdissection of the mouse kidney that TLR5 is expressed in renal tubules, mostly in the distal tubules and in the collecting duct cells while almost not expressed in the proximal tubules (13). However, although Copenhageni Fiocruz L1-130 is the only strain recognized by mouse TLR5 upon destabilization by heating (18, 47), we did not find more leptospires in kidneys of TLR5ko mice compared to WT mice. This result is also in agreement with the fact that treatment with antimicrobial peptides on live leptospires did not unmask a mouse TLR5 signal. Therefore, we may hypothesize that the localization of leptospires in proximal tubules, apart from being the first place to be reached by leptospires and potentially providing rich surroundings for nutrients, could also constitute a favorable environment to avoid the innate TLR5 response in other animals.

Our results also highlighted an important feature of bovine immune response toward leptospires, since we showed that bovine TLR5 recognizes heated and *Leptospira* treated with antimicrobial peptides.

Since antimicrobial peptides affect live leptospires allowing for TLR5 recognition and signaling, and because bovine antimicrobial peptides are potent to kill leptospires (37), our results suggest that the bovine TLR5 response may be important to fight leptospires in cattle. Interestingly, bovine TLR5 has been described to present bacterial species-specificity of flagellin recognition (27, 48). In our study, the magnitude of the *Leptospira*-induced bovine TLR5 signaling was intermediate between the weak response observed with murine TLR5 and the response seen with human TLR5 (Figure 5C). However, rather than reflect real differences between bovine and human TLR5, this lower response may actually result from the heterologous expression system of bovine TLR5 in the human HEK cell system, that has been shown to impact the responsiveness (48). Together, we speculate that these observed differences in TLR5 sensing between animals and also between the three strains of *L. interrogans* tested, could, at least partly, be responsible for shaping the preferential species-specificity adaptation of *Leptospira* serovars to their hosts (49).

Our results showing the lack of TLR5 signaling by live bacteria despite the involvement of the FlaB subunits in the TLR5 recognition could have been anticipated considering the peculiarities of the leptospiral endoflagella. Indeed, the recent published structure of the filament of the leptospiral flagella showed that the FlaBs form the core and are wrapped inside a lattice composed of both FlaAs, FcpA and FcpB subunits (8) therefore hiding the FlaB monomers. The localization of the flagella inside the periplasm adds another additional layer of protection from the host innate immune system. In addition, in *Enterobacteriaceae*, a unique FliC monomer polymerizes to form 11 protofilaments that together assemble to constitute one flagellum filament. The consensus sites for TLR5 recognition in the flagellin FliC are localized at stacking sites between the flagellin monomers and therefore are not accessible when the filament is formed. Hence, when polymerized, the interaction domain of FliC with TLR5 is masked, therefore whole flagella do not signal through TLR5, which occurs only when FliC is monomeric (41, 50, 51). Intact purified periplasmic flagella from *Treponema denticola* were not able to activate TLR5 as well (52). Interestingly, our data suggest that, similar to *Enterobacteriaceae* (10), the leptospiral flagellum depolymerizes at 70°C, which would allow for the release of monomers recognized by the human TLR5. However, in contrast with the *Salmonella* FliC, which is inactivated after 15 min at 100°C, leptospiral flagellins in the context of whole flagella appear to be highly resistant to heating.

Interestingly, we also showed a very high stability of the leptospiral filaments and FlaB proteins that resist heating to 100°C for 30 min and 85°C for 3 h. This unusual thermoresistance of the leptospiral flagella is reminiscent of the hydrophobic and very highly glycosylated pili of hyperthermophilic *Archaea* (53). Glycosylations also occur in bacteria. Although we do not know whether the *Treponema* FlaBs are particularly stable, it has recently been shown that the FlaBs of *Treponema denticola* were glycosylated with an unusual novel glycan (54). Mass spectrometry analysis of these glycopeptides revealed FlaBs glycosylation by O-linkage at multiple sites near the D1 domain, in the very conserved region that interacts with TLR5 (encompassing the end of consensus 2) (Supplementary Figure 6A) (54). Interestingly, we found that these atypical glycosylations target sequences in *Treponema*, notably the two motifs “VEV**S**QL” and “DRIA**S**” are almost 100% conserved in the FlaB1, FlaB2 and FlaB3 of pathogenic and saprophytic *Leptospira* (Supplementary Figures 6B,C) (54). In addition, this consensus was also 100% conserved in leptospiral FlaB1 from other major species involved in leptospirosis in animals and humans (Supplementary Figure 6D). Interestingly, the two serine residues were substituted in the *L. interrogans* FlaB4 and FlaB from *Borrelia burgdorferi* (Supplementary Figure 6C), which might suggest a lack of glycosylation of the leptospiral FlaB4 subunit and *B. burgdorferi* FlaB. The authors hypothesized that in *Treponema* spp. these peculiar glycosylations could impair the TLR5 signaling of *Treponema*. Our study suggests, if these post-translational modifications exist in leptospires, that they would not impair the TLR5 recognition at least in human and bovine TLR5. Rather we may speculate that they could participate in the thermoresistance of the filament structure.

In other spirochetes, the filament structure differs from the leptospiral one since in *Treponema* and *Borrelia* spp. the FcpA subunits are absent, and beside, in *Borrelia* only one copy of FlaA and FlaB compose the filament (55, 56). The stability of the leptospiral filament is most probably due to the particular association and spatial arrangement of the different FlaBs and to their recently described asymmetric interactions with FlaAs or with Fcps (8). Whether the four FlaBs are randomly dispersed along the filament or would have specific structural functions remains to be studied. However, our results were obtained in the context of the whole bacteria. It would have been interesting to test individual leptospiral FlaB subunits to understand whether the high stability results from intrinsic properties of the individual FlaBs. However, our attempts to express recombinant FlaB monomers have failed. We cannot exclude a caveat in our cloning strategy but this failure was quite surprising considering that *T. denticola* and *T. pallidum* FlaB were expressed as stable recombinant proteins that were able to signal through TLR5 in THP1 monocytes or in human keratocytes, respectively (42). One hypothesis could be that the FlaBs that encompass a different shape than FliC would need to be stabilized by polymerization into the complex filament structure.

The respective role of the leptospiral FlaB1, FlaB2, FlaB3 and FlaB4 proteins remains unknown. The Phyre 2 models suggest that the four FlaBs structures are identical, which explains why the precise roles of the different FlaBs in the core could not be addressed in a recent structural study (8). The only information available about differences in the four subunits comes from a proteomic study (7) that finds all four FlaB subunits in Fiocruz L1-130 strain cultured in EMJH at 30°C, suggesting that all subunits were present in the filaments with different relative abundance of FlaB subunits. Each bacterium contained 12,000 copies of FlaB1, 2,000 copies of FlaB2, 300 copies of FlaB3 and 3,500 copies of FlaB4 (7). We tested the expression of each of the four FlaBs mRNA in EMJH cultures and found that all the subunits were expressed in the Icterohaemorrhagiae Verdun and Copenhageni Fiocruz L1-130 strains. However, the relative mRNA levels of the different FlaB subunits did not match the data obtained in the proteomic study, since for example the relative mRNA quantities of *flaB3* seems to be higher than *flaB4* at the stationary phase. Of note the *flaB3* mRNA expression was upregulated at the stationary phase in the 3 serovars of *L. interrogans*, for which we have no explanation. Of note, the *flaB1* expression was upregulated at the stationary phase in Fiocruz L1-130, which could potentially explain the striking difference between the Manilae L495 strain that was not recognized by the mouse TLR5 whereas the Copenhageni L1-130 strain exhibited a better recognition, despite the fact that all their FlaBs are almost identical and 100% conserved in the TLR5 consensus binding domains. In addition, the absence of one FlaB subunit in the *flaB4* mutant of *L. biflexa* Patoc I, which has been shown to impair the filament formation (19), also impairs the TLR5 signaling. A decreased TLR5 signaling was also observed with the Manilae *flaB1* mutant although the impact of this mutant on filament formation has not yet been studied. However, in both cases the TLR5 signal was not abolished, suggesting that despite the lack of observed motility and filaments, some other FlaB subunits were still expressed and able to signal through TLR5, in agreement with the *in silico* analyses suggesting that all FlaB subunits can in theory signal through TLR5.

The fact that we found a striking down-regulation of FlaBs, not of FlaAs, in blood of mice and hamsters 24 h p.i with the Icterohaemorrhagiae Verdun strain, suggests that a regulation of the FlaBs expression could favor an escape from the TLR5 immune surveillance upon infection. However, it remains to be demonstrated that the global down-regulation of FlaBs expression that we observed *in vitro* at the exponential phase correlates indeed with a decrease in TLR5 recognition. In animal blood, the downregulation of the FlaB expression could make sense to avoid the TLR5 response. It would have been interesting to check the expression of the FlaBs in *Leptospira* colonizing the kidney of animals. However, if amenable in the blood of animals, the purification of leptospires mRNA in kidneys is still challenging. The only example of published renal transcriptome dualseq analysis of *L. interrogans* (Fiocruz L1-130) infection in mice could only detect 29 leptospiral genes (57), among them *lipL32*, encoding the major lipoprotein and interestingly, one flagellin gene, *flaB4* (LIC11531), suggesting that the mRNA levels of FlaB4 were quite high, and potentially higher than the other FlaBs mRNA. As a whole, these results suggest a complex regulation of the leptospiral FlaB subunits that deserves further investigation. Interestingly, it was shown in another spirochete *Brachyspira hyodysenteriae* that the flagellin genes are transcribed by different transcription factors, with sigma 28 regulating the *flaB1* and *flaB2* genes, whereas the *flaA* and *flaB3* genes are controlled by sigma 70. The authors suggest that the relative ratio of the flagellin proteins could play a role in the stiffness of the flagellar filament and consequently that this regulation may play a role in motility (58). The regulation of FlaBs in leptospires that harbor an even more complex flagellar filament is an interesting question that remains to be studied. Our findings of *in vivo* downregulation of the FlaBs deserve further studies potentially linking it to regulation of *Leptospira* motility *in vivo*.

Interestingly, the leptospiral FlaBs share with the flagellin of *Bacillus* spp., that is also able to signal via TLR5, a similar structure made of the D0 and D1 domains of FliC and lacking the D2 and D3 domains (59). Of note, the D2 and D3 domains of FliC are highly variable and responsible for the strong antigenicity of flagellins in *Enterobacteriaceae* (10). Flagellin is known to be a potent vaccine adjuvant, however, the antigenicity of the D2 and D3 domains can be a problem when booster immunizations are done. To circumvent this issue, several strategies have been recently proposed. The first consisted in using a FliC devoid of the D2 and D3 domains (14), and the second to use the *Bacillus* flagellin as an expression platform (59). Likewise, we may speculate in the case of *Leptospira* spp. that upon *in vivo* killing and exposure of FlaB subunits, the lack of D2 and D3 domains could be advantageous to limit the antibody response. Hence, the peculiar structure of FlaBs could also participate in the adaptive immune evasion.

In conclusion, we showed here that pathogenic *Leptospira* largely escape recognition by TLR5. Other bacteria such as *Helicobacter pylori* have been shown to escape the TLR5 response through modification of the amino residues in the D0 or D1 regions of flagellin subunits (43), but leptospires seem to differ in avoiding TLR5 recognition. Indeed, our data demonstrate that the endoflagella play a role in the escape from TLR5 surveillance, which has never been shown before and might hold true for other spirochetes. We also evidenced regulatory mechanisms of *flaB* genes expression that may also play a role in this immune evasion and have important consequences since TLR5 ligation has a potent adjuvant role in immunity.

## Data Availability Statement

All datasets presented in this study are included in the article/Supplementary Material.

## Ethics Statement

The animal study was reviewed and approved by (#2013-0034 and #HA-0036) the Institut Pasteur ethic committee (CETEA #89) (Paris, France), the competent authority, for compliance with the French and European (EU directive 2010/63) regulations on Animal Welfare and with Public Health Service recommendations.

## Author Contributions

CW: conception, project administration, supervision, and writing original draft. MH, DB, JC, MM, and CW: visualization. MH, DB, JC, FV-P, SB, MF, MM, CG, and CW: investigation and data analysis. MF and FV-P: validation. LF, FV-P, and MM: methodology. DB and SB: sequences alignment. MP, EW, AK, and DW: resources. CG, EW, IB, and CW: funding acquisition. DW and IB: English editing. All authors contributed to the writing, review and editing of the manuscript, and approved the submitted version.

## Conflict of Interest

The authors declare that the research was conducted in the absence of any commercial or financial relationships that could be construed as a potential conflict of interest.

## Supplementary Material

FIGURE S1 Not antibiotics but antimicrobial peptides reveal the L495 ability to activate TLR5. **(A)** NF-κB reporter assay in HEK-Blue-KD-TLR5 cells transfected with the human TLR5 (blue bars), or empty plasmid (empty bars) and stimulated with MOI 100 of *L. interrogans* Manilae strain L495 treated with gentamicin (50 μg/mL), penicillin G (10 μg/mL), azithromycin (10 μg/mL) or daptomycin (1 μg/mL) for 4 h before stimulation. Data are expressed as the mean (±SD) of technical replicates (*n* = 3). **(B)** NF-κB reporter assay in HEK-Blue-KD-TLR5 cells transfected with the bovine TLR5 (dark blue bars), mouse TLR5 (light blue bars) or empty plasmid (empty bars) and stimulated with MOI 100 of *L. interrogans* Manilae strain L495 treated with human peptide LL-37 or bovine peptide Bmap28 at various concentration (0–250 μg/mL) for 2 h before stimulation. Unpurified Fla from *Salmonella typhimurium* (500 ng/mL) was used as control. Data are expressed as the mean (±SD) of technical replicates (*n* = 3) and are representative of at least three independent experiments. Statistically significant differences (Student *t*-test) are indicated.

FIGURE S2 Schematics of leptospiral filament and FliC association with TLR5. Schematic representations of **(A)** the leptospiral flagellin structure adapted from Gibson et al. (8) and **(B)** the interaction of FliC subunits with TLR5 inducing dimerization of the receptors adapted from Yoon et al. (40).

FIGURE S3 BLAST analyses between flagellin subunits and species. **(A–C)** Amino acid sequence homology percentage between (A) *Leptospira interrogans* strain Fiocruz FlaBs (LIC11531, LIC18890, LIC11889, LIC11532) and FlaAs (LIC10788, LIC10787), **(B)** *Leptospira interrogans* strain Fiocruz FlaBs or *Leptospira biflexa* strain Patoc FlaBs (LEPBIa1589, LEPBIa2133, LEPBIa2132, LEPBIa1872), **(C)** *Leptospira interrogans* FlaB1 of all serotypes (Fiocruz LIC11531, Manilae LMANv2_590023, Verdun AKWP_v1_110067, Patoc LEPBIa1589), FlaB2 of all serotypes (Fiocruz LIC11890, Manilae LMANv2_260016, Verdun AKWP_v1_110429, Patoc LEPBIa2133), FlaB3 of all serotypes (Fiocruz LIC11889, Manilae LMANv2_260015, Verdun AKWP_v1_110428, Patoc LEPBIa2132) and FlaB4 of all serotypes (Fiocruz LIC11532, Manilae LMANv2_590024, Verdun AKWP_v1_110068, Patoc LEPBIa1872).

FIGURE S4 TLR5 binding and consensus sites in different species. Clustal (MEGA software) alignment of the amino acid sequences for the TLR5 binding consensus regions of: *Leptospira interrogans* strain Fiocruz FlaB1 (LIC11531), *Leptospira biflexa* strain Patoc FlaB1 (LEPBIa1589), *Borrelia burgdorferi* (GeneBank CAA45011.1), *Treponema* ssp. (GeneBank AIW88993.1), *Bacillus subtilis* strain W23 (GeneBank ADM39502.1), *Salmonella enterica* subsp. *enterica* serovar Typhimurium (GeneBank QDQ31983.1), *Escherichia coli* strain 0157:H7 (KKF82802.1), *Helicobacter pylori* strain J99 (GeneBank AKE81874.1) and *Bartonnela bacilliformis* (GeneBank AAA22899.1).

FIGURE S5 Temperature of culture medium does not alter FlaAs and FlaBs regulation. FlaAs and FlaBs mRNA expression in culture in EMJH at 30 or 37°C. Data of RT-qPCR are expressed as the ratio of mRNA quantities relatives to the EMJH 30°C control. Technical replicates are represented as dots and lines correspond to mean (±SD) of all replicates.

FIGURE S6 Glycosylation sites on FlaBs. **(A,B)** *In silico* (Phyre2 and Chimera softwares) prediction of **(A)** *Treponema pallidum* strain Nichols FlaB1 (P21990) and **(B)** *Leptospira interrogans* strain Fiocruz FlaB1 (LIC11531) with TLR5 binding consensus 2 (yellow) and potential glycosylation positions (pink) highlighted. **(C)** Clustal (MEGA software) alignment of the amino acid sequences for region with potential glycosylations of: *Treponema denticola* FlaB (GeneBank WP_010697276.1), *Borrelia burgdorferi* (GeneBank CAA45011.1), *L. biflexa* (strain Patoc) FlaB1 (LEPBIa1589), FlaB2 (LEPBIa2133), FlaB3 (LEPBIa2132), FlaB4 (LEPBIa1872), *L. interrogans* (strain Fiocruz) FlaB1 (LIC11531), FlaB2 (LIC18890), FlaB3 (LIC11889), FlaB4 (LIC11532), *L. interrogans* (strain Manilae) FlaB1 (LMANv2_590023), FlaB2 (LMANv2_260016), FlaB3 (LMANv2_260015), FlaB4 (LMANv2_590024) and *L. interrogans* (strain Verdun) FlaB1 (AKWP_v1_110067), FlaB2 (AKWP_v1_110429), FlaB3 (AKWP_v1_110428) and FlaB4 (AKWP_v1_110068).
