# Corrigendum: Escape of TLR5 recognition by *Leptospira* spp.: A rationale for atypical endoflagella

**DOI:** 10.3389/fimmu.2022.932151

**Published:** 2022-11-08

**Authors:** Marion Holzapfel, Delphine Bonhomme, Julie Cagliero, Frédérique Vernel-Pauillac, Martine Fanton d’Andon, Sophia Bortolussi, Laurence Fiette, Cyrille Goarant, Elsio A. Wunder, Mathieu Picardeau, Albert I. Ko, Dirk Werling, Mariko Matsui, Ivo G. Boneca, Catherine Werts

**Affiliations:** ^1^Institut Pasteur, Unité Biologie et Génétique de la Paroi Bactérienne, Paris, France; ^2^CNRS, UMR 2001 Microbiologie Intégrative et Moléculaire, Paris, France; ^3^Institut National de la Santé et de la Recherche Médicale, Equipe Avenir, Paris, France; ^4^Sorbonne Paris Cité, Université de Paris, Paris, France; ^5^Institut Pasteur de Nouvelle Calédonie, Immunity and Inflammation Group, Institut Pasteur International Network, Noumea, France; ^6^Unité Histopathologie Humaine et Modèles Animaux, Institut Pasteur, Paris, France; ^7^Leptospirosis Research and Expertise Unit, Institut Pasteur International Network, Institut Pasteur de Nouvelle Calédonie, Noumea, France; ^8^Gonçalo Moniz Institute, Oswaldo Cruz Foundation, Brazilian Ministry of Health, Salvador, Brazil; ^9^Department of Epidemiology of Microbial Diseases, Yale School of Public Health, New Haven, CT, United States; ^10^Unité Biologie des Spirochètes, Institut Pasteur, Paris, France; ^11^Department of Pathobiology and Population Sciences, Royal Veterinary College, Hatfield, United Kingdom

**Keywords:** Leptospira, toll-like receptor, innate immunity, Flagelin genes, TLR5, mouse model

In the original article, there was a mistake in **Figure 8A**, as published. We showed that we did not get expression of the FLaB1 subunit in Manilae L495 strain. In fact, the forward primer (designed according to the Fiocruz sequence) used to amplify the FlaB1 subunit has two mismatches within the Manilae sequence. We did the RT-PCR with the good primer and found an amplification, showing an enhanced expression at the stationary phase compared to the exponential phase, likewise the other FlaB subunits**.** The corrected **Figure 8** appears below.

A correction has been made to **Results**, “*FlaB mRNA Are Upregulated in Stationary Phase”*.

**“**Of note, and different from other strains, the Manilae L495 flaB1 mRNA was undetectable***”*** and a paragraph in the **Discussion**, **“**Furthermore, in Manilae L495…**”** are not accurate and should be both omitted.

In the original article, there was also mistake in **Figure 6**, **Supplementary Figure 3**, **Supplementary Figure 4** and **Supplementary Figure 6** and their legends as published. We realized a “slipping” of the names of FlaB subunits only in the figures of alignments and comparison of sequences (**Figure 6**, **Sup Figure 3**, **Sup Figure 4** and **Sup Figure 6**). What we called FlaB1 in figures and respective legends is in fact FlaB4, FlaB2 is FlaB1, FlaB3 is FlaB2, and FlaB4 is FlaB3 (see corrected annotation in **Additional table** provided).

**Figure 6:**
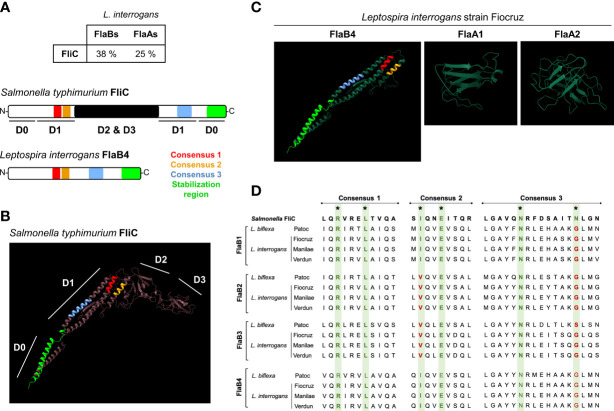
**(A)** Amino acid sequence homology average percentage between *Salmonella typhimurium* FliC (P06179) and *Leptospira interrogans* strain Fiocruz FlaB (LIC11890, LIC11889, LIC 11532 and LIC11531) and FlaAs (LIC10788 and LIC10787) and primary structures of the flagellin proteins with TLR5 binding consensus. **(B)** *In silico* (Phyre2 and Chimera softwares) prediction of *Salmonella typhimurium* FliC (P06179) structure with the four described domains and with positions of the TLR5 binding consensus: 1 (red), 2 (yellow) and 3 (light blue) and stabilization region (light green) highlighted. **(C)** *In silico* (Phyre2 and Chimera softwares) prediction of *Leptospira interrogans* strain Fiocruz FlaB4 (LICI1531) with the positions of the TLRS binding consensus and stabilization region highlighted, FlaA1 (LIC10788), FlaA2 (LICI0787). **(D)** Clustal (MEGA software) alignment of the amino acid sequences for the TLR5 binding consensus regions of: *Salmonella enterica* FliC (GeneBank QDQ31983.1), *L. bifleva* (strain Patoc) FlaB1 (LEPBla2133), FlaB2 (LEPBIa2132), FlaB3 (LEPBla1872) and FlaB4 (LEPBla1589), L. interrogans (strain Fiocruz L1-130) FlaB1 (LIC18890), FlaB2 (LICIT889), FlaB3 (LICI1532) and FlaB4 (LIC11531), *L. interrogans* (strain L495) FlaB1 (LMANv2 260016), FlaB2 (LMANv2 260015). FlaB3 (LMANv2 590024) and FlaB4 (LMANv2 590023), and L. interrogans (strain Verdun) FlaB1 (AKWP_v1_110429), FlaB2 (AKWP_v1_110428) and FlaB3 (AKWP_v1_110068) and FlaB4 (AKWP_v1_110067).

**Table:** Additional Table Old annotations (published Frontiers Immunol 2020).

10788	Patoc	Fiocruz L1-130	Manilae	Verdun
*flaA1*	LEPBIa2335	LIC10788	LMANv2_260046	AKWP_v1_210009
*flaA2*	LEPBIa2336	LIC10787	LMANv2_260045	AKWP_v1_210008
*flaB1*	LEPBIa1589	LIC11531	LMANv2_590023	AKWP_v1_110067
*flaB2*	LEPBIa2133	LIC11890	LMANv2_260016	AKWP_v1_110429
*flaB3*	LEPBIa2132	LIC11889	LMANv2_260015	AKWP_v1_110428
*flaB4*	LEPBIa1872	LIC11532	LMANv2_590024	AKWP_v1_110068

**Table:** New annotations consistent with studies from M Picardeau.

10788	Patoc*	Fiocruz L1-130*	Manilae*	Verdun*
*flaA1*	LEPBIa2335	LIC10788	LMANv2_260046 / LIMLP_13775	AKWP_v1_210009
*flaA2*	LEPBIa2336	LIC10787	LMANv2_260045 / LIMLP_13780	AKWP_v1_210008
*flaB1*	LEPBIa2133	LIC11890	LMANv2_260016 / LIMLP_09410	AKWP_v1_110429
*flaB2*	LEPBIa2132	LIC11889	LMANv2_260015 /LIMLP_09405	AKWP_v1_110428
*flaB3*	LEPBIa1872	LIC11532	LMANv2_590024 / LIMLP_07480	AKWP_v1_110068
*flaB4*	LEPBIa1589	LIC11531	LMANv2_590023 / LIMLP_07475	AKWP_v1_110067

*Annotation of *L. biflexa* serovar Patoc strain Patoc 1 (Patoc), *L. interrogans* serovar Copenhageni strain Fiocruz L1-130 (Fiocruz L1-130), *L. interrogans* serovar Manilae strain L495 (Manilae), *L. interrogans* serovar Manilae UP-MMC-NIID LP (Manilae) (ref Satou et al. 2015 ; PMID: 26272567).

*L. interrogans* serovar Icterohaemorrhagiae Verdun LP (Verdun) in https://mage.genoscope.cns.fr/microscope/home/index.php.

However, the annotations used in **Figures 7**, **8** were correct and all the mutants used are correct. The corrected **Figure 6** and its corrected legend appears below. The corrected **Supplementary Figures 3**, **4**, and **6** can be accessed from the original article.

**Figure 8:**
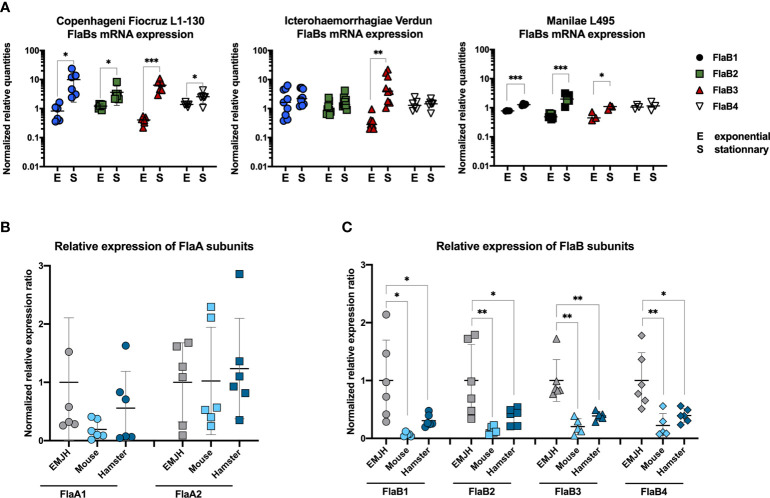
**(A)** *In vitro* FlaBs mRNA expression in *L. interrogans* Copenhageni Fiocruz L1-130, Icterohaemorrhagiae Verdun and Manilae L495 at the exponential (E) and stationary (S) phase. Data of RT-qPCR are expressed as the relative mRNA quantities normalized to the expression of the lipl41 mRNA. Technical replicates are represented as dots and lines correspond to mean (+/- SD) of replicates (3 < n < 9). Statistically significant differences (Student t-test) are indicated. **(B)** In vivo FlaAs and **(C)** FlaBs mRNA expression in blood of infected mice (n=5, light blue) and hamsters (n-5, dark blue), 24 h post intraperitoneal infection with 2x108 virulent *L. interrogans* Icterohaemorrhagiae strain Verdun, compared with mRNA expression in culture in EMJH at 30° C. Data of RT-gPCR are expressed as the ratio of mRNA quantities relatives to the EMJH control. Individual animals are represented as dots and lines correspond to mean (+/- SD) of all animals. Statistically significant differences (Student t-test) are indicated with corresponding p values: * for p < 0.05; ** for p < 0.01 and *** for p < 0.001.

The authors apologize for these errors and state that they do not change the scientific conclusions of the article in any way. The original article has been updated.

## Publisher’s note

All claims expressed in this article are solely those of the authors and do not necessarily represent those of their affiliated organizations, or those of the publisher, the editors and the reviewers. Any product that may be evaluated in this article, or claim that may be made by its manufacturer, is not guaranteed or endorsed by the publisher.

