# Supplementary material for: Escape of TLR5 Recognition by Leptospira spp.: A Rationale for Atypical Endoflagella
Source: Front Immunol. 2020 Aug 11;11:2007. doi: 10.3389/fimmu.2020.02007 (PMC7431986; doi:10.3389/fimmu.2020.02007)
Supplement: FIGURE S1 — Not antibiotics but antimicrobial peptides reveal the L495 ability to activate TLR5. (A) NF-κB reporter assay in HEK-Blue-KD-TLR5 cells transfected with the human TLR5 (blue bars), or empty plasmid (empty bars) and stimulated with MOI 100 of L. interrogans Manilae strain L495 treated with gentamicin (50 μg/mL), penicillin G (10 μg/mL), azithromycin (10 μg/mL) or daptomycin (1 μg/mL) for 4 h before stimulation. Data are expressed as the mean (±SD) of technical replicates (n = 3). (B) NF-κB reporter assay in HEK-Blue-KD-TLR5 cells transfected with the bovine TLR5 (dark blue bars), mouse TLR5 (light blue bars) or empty plasmid (empty bars) and stimulated with MOI 100 of L. interrogans Manilae strain L495 treated with human peptide LL-37 or bovine peptide Bmap28 at various concentration (0–250 μg/mL) for 2 h before stimulation. Unpurified Fla from Salmonella typhimurium (500 ng/mL) was used as control. Data are expressed as the mean (±SD) of technical replicates (n = 3) and are representative of at least three independent experiments. Statistically significant differences (Student t-test) are indicated. [file Data_Sheet_1.pdf]

Sup Figure 1. **Not antibiotics but antimicrobial peptides reveal the L495 ability to activate TLR5**

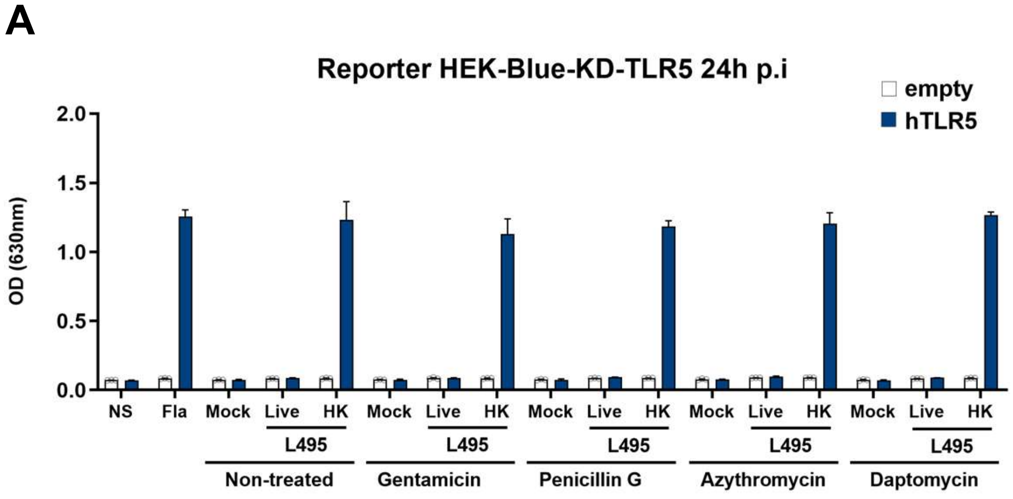

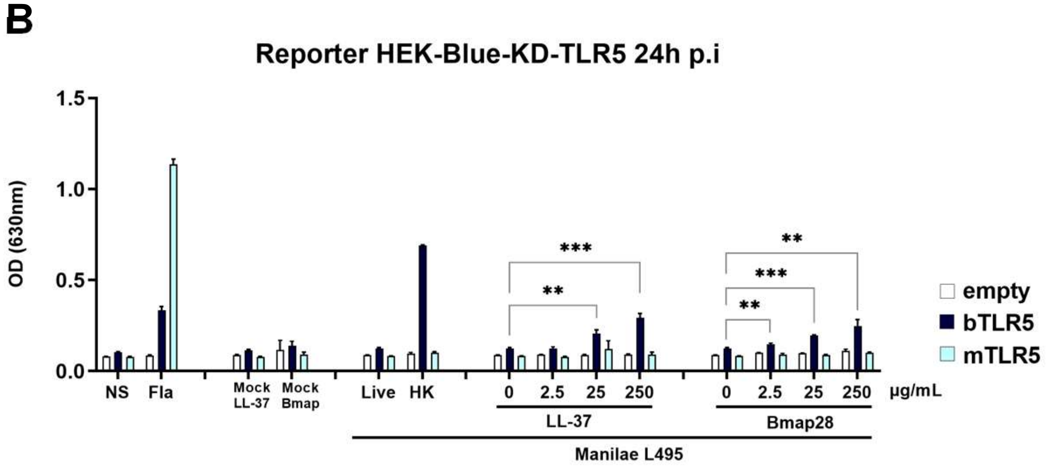

Sup Figure 1. Not antibiotics but antimicrobial peptides reveal the L495 ability to activate TLR5

A) NF-κB reporter assay in HEK-Blue-KD-TLR5 cells transfected with the human TLR5 (blue bars), or empty plasmid (empty bars) and stimulated with MOI 100 of *L. interrogans* Manilae strain L495 treated with gentamicin (50 µg/mL), penicillin G (10 µg/mL), azithromycin (10 µg/mL) or daptomycin (1 µg/mL) for 4 h before stimulation. Data are expressed as the mean (+/- SD) of technical replicates (n= 3). **B)** NF-κB reporter assay in HEK-Blue-KD-TLR5 cells transfected with the bovine TLR5 (dark blue bars), mouse TLR5 (light blue bars) or empty plasmid (empty bars) and stimulated with MOI 100 of *L. interrogans* Manilae strain L495 treated with human peptide LL-37 or bovine peptide Bmap28 at various concentration (0-250 µg/mL) for two hours before stimulation. Unpurified Fla from *Salmonella typhimurium* (500 ng/mL) was used as control. Data are expressed as the mean (+/- SD) of technical replicates (n= 3) and are representative of at least three independent experiments. Statistically significant differences (Student t-test) are indicated.

Sup Figure 2. **Schematics of leptospiral filament and FliC association with TLR5**

A

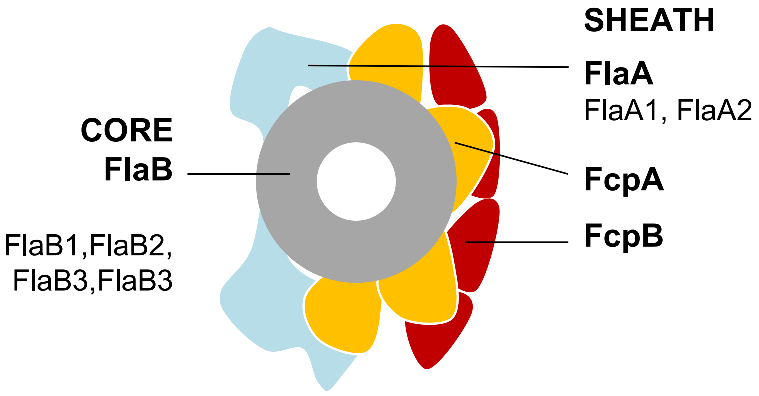

Adapted from « An asymmetric sheath controls flagellar supercoiling and motility in the leptospira spirochete. Gibson *et al.*, *eLife* (2020) »

B

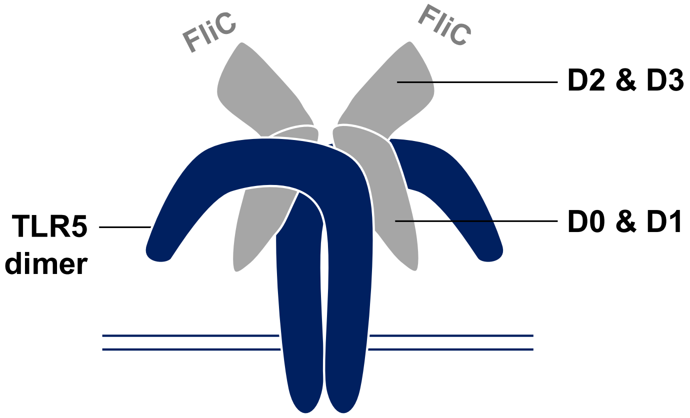

Adapted from « Structural basis of TLR5-Flagellin Recognition and Signaling. Yoon *et al.*, *Science* (2012) »

Sup Figure 2. Schematics of leptospiral filament and FliC association with TLR5

Schematic representations of **A)** the leptospiral flagellin structure adapted from Gibson *et al.* 2020 and **B)** the interaction of FliC subunits with TLR5 inducing dimerization of the receptors adapted from Yoon *et al.* 2012.

Sup Figure 3. BLAST analyses between leptospiral flagellin subunits and species

A

BLAST-P identity % **Fiocruz**	FlaB1	FlaB2	FlaB3
FlaB4	72%	67%	51%
FlaB1	/	72%	55%
FlaB2	/	/	57%

BLAST-P identity % **Patoc**	FlaB1	FlaB2	FlaB3
FlaB4	69%	64%	49%
FlaB1	/	68%	52%
FlaB2	/	/	55%

B

BLAST-P (Identity %) **FlaB1**	Manilae	Verdun	Patoc
**Fiocruz**	100%	100%	62%

BLAST-P (Identity %) **FlaB4**	Manilae	Verdun	Patoc
**Fiocruz**	99%	100%	92%
**Manilae**	/	99%	91%

BLAST-P (Identity %) **FlaB3**	Manilae	Verdun	Patoc
**Fiocruz**	99%	100%	87%
**Manilae**	/	99%	87%

BLAST-P (Identity %) **FlaB2**	Manilae	Verdun	Patoc
**Fiocruz**	100%	100%	78%

Sup Figure 3. BLAST analyses between flagellin subunits and species

A-B) Amino acid sequence homology percentage between **A)** *Leptospira interrogans* strain Fiocruz FlaBs (LIC18890, LIC11889, LIC11532, LIC11531) or *Leptospira biflexa* strain Patoc FlaBs (LEPBIa2133, LEPBIa2132, LEPBIa1872, LEPBIa1589), **B)** *Leptospira interrogans* FlaB1 of all serotypes (Fiocruz LIC11890, Manilae LMANv2_260016, Verdun AKWP_v1_110429, Patoc LEPBIa2133), FlaB2 of all serotypes (Fiocruz LIC11889 , Manilae LMANv2_260015, Verdun AKWP_v1_110428, Patoc LEPBIa2132), FlaB3 of all serotypes (Fiocruz LIC11532, Manilae LMANv2_590024, Verdun AKWP_v1_110068, Patoc LEPBIa1872) and FlaB4 of all serotypes (Fiocruz LIC11531, Manilae LMANv2_590023, Verdun AKWP_v1_110067, Patoc LEPBIa1589).

Sup Figure 4. TLR5 binding and consensus sites in different species

A

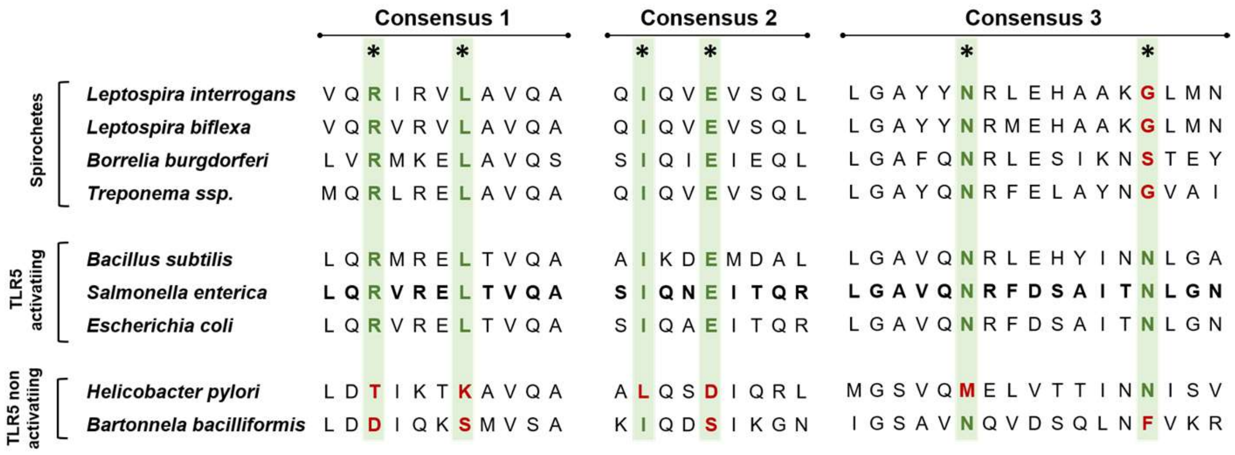

Adapted from « A conserved TLR5 binding and activation hot spot on flagellin. Song *et al.*, *Sci. Reports* (2017) »

B

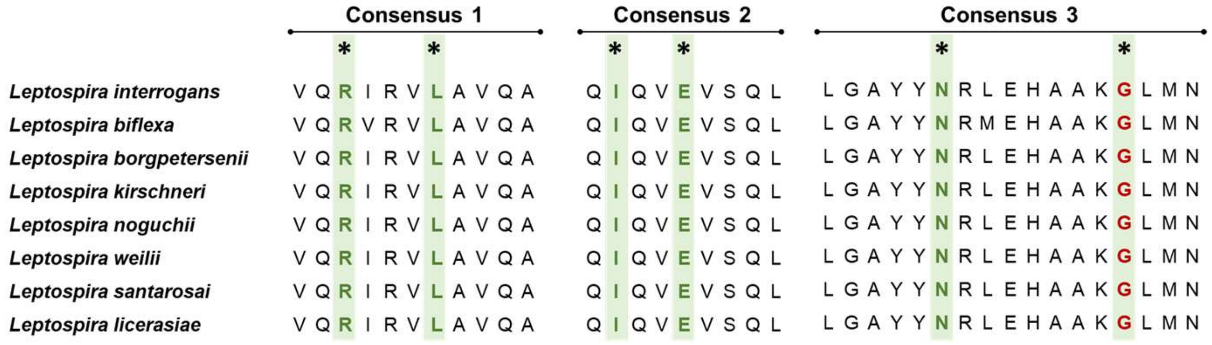

Sup Figure 4. TLR5 binding and consensus sites in different species

A-B) Clustal (MEGA software) alignment of the amino acid sequences for the TLR5 binding consensus regions of: **A)** *Leptospira interrogans* strain Fiocruz FlaB4 (LIC11531), *Leptospira biflexa* strain Patoc FlaB4 (LEPBIa1589), *Borrelia burgdorferi* (GeneBank CAA45011.1), *Treponema* ssp. (GeneBank AIW88993.1), *Bacillus subtilis* strain W23 (GeneBank ADM39502.1), *Salmonella enterica* subsp. *enterica* serovar Typhimurium (GeneBank QDQ31983.1), *Escherichia coli* strain 0157:H7 (KKF82802.1), *Helicobacter pylori* strain J99 (GeneBank AKE81874.1) and *Bartonnela bacilliformis* (GeneBank AAA22899.1) and **B)** FlaB4s or homologs of *L. interrogans* Copenhageni strain Fiocruz L1-130 (LIC11531), *L. biflexa* Patoc strain Patoc (LEPBIa1589), *L. borgpetersenii* Hardjo-bovis strain JB197 (Q04TC0_LEPBJ), *L. kirschneri* strain H1 (A0A0E2B0X3_9LEPT), *L. noguchii* Autumnalis strain ZUN142 (M6UC47_9LEPT), *L. weilii* Topaz strain LT2116 (M3G645_9LEPT), *L. santarosai* Arenal strain MAVJ 401 (M6JKS8_9LEPT) and *L. licerasiae* Varillal strain VAR 010 (I0XRK5_9LEPT).

Sup Figure 5. **Temperature of culture medium does not impact FlaAs and FlaBs regulation**

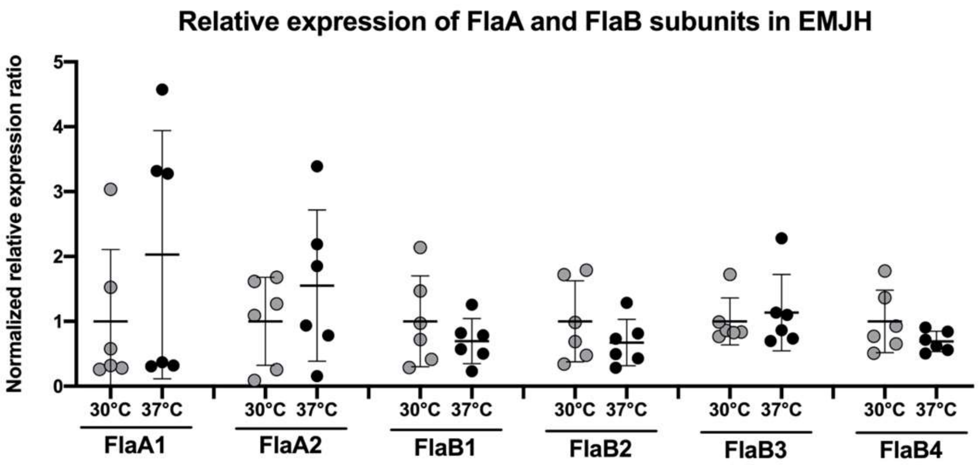

Sup Figure 5. Temperature of culture medium does not alter FlaAs and FlaBs regulation

FlaAs and FlaBs mRNA expression in culture in EMJH at 30° C or 37° C. Data of RT-qPCR are expressed as the ratio of mRNA quantities relatives to the EMJH 30° C control. Technical replicates are represented as dots and lines correspond to mean (+/- SD) of all replicates.

Sup Figure 6. Glycosylation sites on FlaBs

A

Treponema pallidum
FlaB1

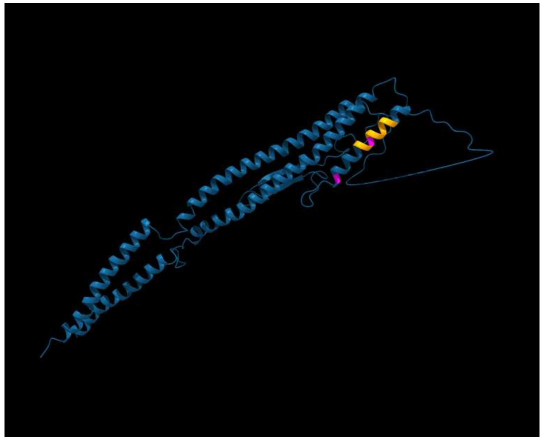

B

Leptospira interrogans
FlaB4

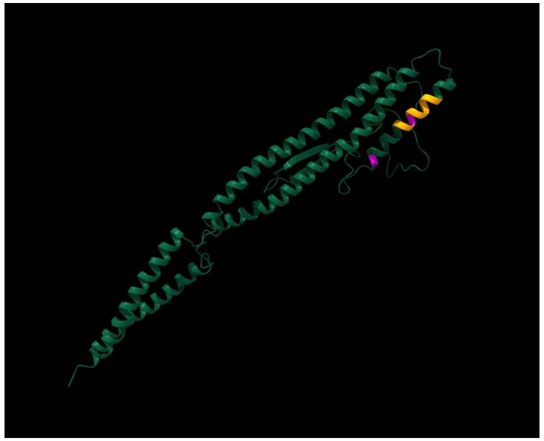

C

Consensus 2

* Glycosylated residues in *Treponema denticola*

			Sequence
Treponema denticola **FlaB1**			**V E V S Q L V A E V D R I A S S**
Borrelia burgdorferi			I E I E Q L T D E I N R I A D Q
FlaB1	*L. biflexa*	Patoc	V E V S Q L I D E V D R I A S Q
	L. interrogans	Fiocruz	V E V S Q L V D E I D R I A S Q
		Manilae	V E V S Q L V D E I D R I A S Q
		Verdun	V E V S Q L V D E I D R I A S Q
FlaB2	*L. biflexa*	Patoc	V E V S A L V D E I D R I A S Q
	L. interrogans	Fiocruz	V E V S A L V D E I D R I A S Q
		Manilae	V E V S A L V D E V D R I A S Q
		Verdun	V E V S A L V D E V D R I A S Q
FlaB3	*L. biflexa*	Patoc	L E V S A L V E E V E R I G T S
	L. interrogans	Fiocruz	L E V D Q L I E E V D R I G K S
		Manilae	L E V D Q L I E E V D R I G K S
		Verdun	L E V D Q L I E E V D R I G K S
FlaB4	*L. biflexa*	Patoc	V E V S Q L V D E I D R I A S Q
	L. interrogans	Fiocruz	V E V S Q L V D E I D R I A S Q
		Manilae	V E V S Q L V D E I D R I A S Q
		Verdun	V E V S Q L V D E I D R I A S Q

D

Consensus 2

* Glycosylated residues in *Treponema denticola*

			Sequence
FlaB4	*L. biflexa*	Patoc	V E V S Q L V D E I D R I A S Q
	L. interrogans	Fiocruz	V E V S Q L V D E I D R I A S Q
		Manilae	V E V S Q L V D E I D R I A S Q
		Verdun	V E V S Q L V D E I D R I A S Q
	L. borgpetersenii		V E V S Q L V D E I D R I A S Q
	L. kirschneri		V E V S Q L V D E I D R I A S Q
	L. noguchii		V E V S Q L V D E I D R I A S Q
	L. weilii		V E V S Q L V D E I D R I A S Q
	L. santarosai		V E V S Q L V D E I D R I A S Q
	L. licerasiae		V E V S Q L V D E I D R I A S Q

Sup Figure 6. Glycosylation sites on FlaBs

A-B) *In silico* (Phyre2 and Chimera softwares) prediction of **A)** *Treponema pallidum* strain Nichols FlaB1 (P21990) and **B)** *L.interrogans* Copenhageni strain Fiocruz L1-130 FlaB4 (LIC11531) with TLR5 binding consensus 2 (yellow) and potential glycosylation positions (pink) highlighted. **C-D)** Clustal (MEGA software) alignment of the amino acid sequences for region with potential glycosylations of: **C)** *Treponema denticola* FlaB1 (GeneBank WP_010697276.1), *Borrelia burgdorferi* (GeneBank CAA45011.1), *L. biflexa* Patoc strain Patoc FlaB1 (LEPBIa2133), FlaB2 (LEPBIa2132), FlaB3 (LEPBIa1872), FlaB4 (LEPBIa1589), *L. interrogans* Copenhageni strain Fiocruz L1-130 FlaB1 (LIC18890), FlaB2 (LIC11889), FlaB3 (LIC11532), FlaB4 (LIC11531), Manilae strain L495 FlaB1 (LMANv2_260016), FlaB2 (LMANv2_260015), FlaB3 (LMANv2_590024), FlaB4 (LMANv2_590023), and Icterohaemorrhagiae strain Verdun FlaB1 (AKWP_v1_110429), FlaB2 (AKWP_v1_110428) and FlaB3 (AKWP_v1_110068), FlaB4 (AKWP_v1_110067), and **D)** FlaB4s or homologs of *L. biflexa* Patoc strain Patoc (LEPBIa1589), *L. interrogans* Copenhageni strain Fiocruz L1-130 (LIC11531), Manilae strain L495 (LMANv2_590023), Icterohaemorrhagiae strain Verdun (AKWP_v1_110067), *L. borgpetersenii* Hardjo-bovis strain JB197 (Q04TC0_LEPBJ), *L. kirschneri* strain H1 (A0A0E2B0X3_9LEPT), *L. noguchii* serovar Autumnalis strain ZUN142 (M6UC47_9LEPT), *L. weilii* serovar Topaz strain LT2116 (M3G645_9LEPT), *L. santarosai* serovar Arenal strain MAVJ 401 (M6JKS8_9LEPT) and *L. licerasiae* serovar Varillal strain VAR 010 (I0XRK5_9LEPT).
